# Uncovering viral RNA–host cell interactions on a proteome-wide scale

**DOI:** 10.1016/j.tibs.2021.08.002

**Published:** 2022-01

**Authors:** Louisa Iselin, Natasha Palmalux, Wael Kamel, Peter Simmonds, Shabaz Mohammed, Alfredo Castello

**Affiliations:** 1 Nuffield Department of Medicine, Peter Medawar Building for Pathogen Research, University of Oxford, Oxford, OX1 3SY, UK; 2 Department of Biochemistry, University of Oxford, South Parks Road, OX1 3QU, Oxford, UK; 3 MRC-University of Glasgow Centre for Virus Research, 464 Bearsden Road, Glasgow G61 1QH, Scotland, (UK); 4 Chemistry Research Laboratory, University of Oxford, Mansfield Road, Oxford, OX1 3TA, UK; 5 The Rosalind Franklin Institute, Oxfordshire, OX11 0FA, UK

**Keywords:** RNA, virus, RNA-binding proteins, proteomics, virus–host interactions, viral ribonucleoprotein

## Abstract

RNA viruses interact with a wide range of cellular RNA-binding proteins (RBPs) during their life cycle. The prevalence of these host–virus interactions has been highlighted by new methods that elucidate the composition of viral ribonucleoproteins (vRNPs). Applied to 11 viruses so far, these approaches have revealed hundreds of cellular RBPs that interact with viral (v)RNA in infected cells. However, consistency across methods is limited, raising questions about methodological considerations when designing and interpreting these studies. Here, we discuss these caveats and, through comparing available vRNA interactomes, describe RBPs that are consistently identified as vRNP components and outline their potential roles in infection. In summary, these novel approaches have uncovered a new universe of host–virus interactions holding great therapeutic potential.

## Highlights

RNA viruses interact with many RNA-binding proteins (RBPs) over the course of their life cycle in both pro- and antiviral capacities.
The true extent of viral (v)RNA–host RBP interactions has only recently become clear as a result of developments in mass spectrometry and methods for specifically studying RBPs.
There are now several methods available for directly studying the vRNA interactome; these use different approaches to specifically capture proteins interacting with vRNA and often yield very different results.
When well designed and correctly interpreted, these methods have the potential to greatly enhance our understanding of viral infection by providing an unbiased insight into viral ribonucleoprotein composition.
Proteins identified in the vRNA interactomes of multiple viruses might have panviral regulatory roles and could be promising targets for developing broad-spectrum antiviral therapies.

## The importance of cellular RNA-binding proteins in virus infection

Pathogenic RNA viruses pose important public health and socioeconomic challenges. As obligate intracellular parasites, viruses rely heavily on host cell proteins for their life cycle, and the scope of these host–virus interactions remains under extensive investigation. **Viral (v)RNA** (see Glossary) has a central role in RNA virus infection because it must act as both mRNA and a viral genome. It is therefore unsurprising that vRNA hijacks a plethora of cellular **RNA-binding proteins** (**RBPs**) to promote viral replication [1]. Cellular RBPs are involved in virtually every step of the viral life cycle, including genome replication, viral protein synthesis, and assembly of virus progeny [2., 3., 4., 5.]. Moreover, vRNA is the target of the antiviral innate immune response because it typically contains unusual molecular signatures that can be recognised by specialised RBPs. These pathogen-associated molecular patterns include triphosphate ends, unmethylated caps, sequence biases, and long double-stranded (ds)RNA tracts produced during viral replication [6., 7., 8.]. Through identifying the complement of cellular proteins that interact with vRNA, it will be possible to discover a new universe of crucial host–virus interactions with potential as targets for host-based antiviral therapies.

Our knowledge of cellular RBPs has expanded dramatically in recent years due to the emergence of novel proteomics-based approaches [9., 10., 11., 12., 13., 14.]. RBPs range from proteins with well-established roles in RNA biology that harbour canonical **RNA-binding domains** (**RBDs**) to others that interact with RNA in unconventional ways, many of which do not have well-characterised biological functions [10]. Several canonical RBPs have been mechanistically linked with viral infection [1,5]. However, the recent discovery of hundreds of novel unconventional RBPs suggests that the breadth of interactions that vRNA can establish with the host cell might be broader than previously anticipated [1,5]. As an illustrative example, the E3 ubiquitin ligase TRIM25 participates in the antiviral response mediated by the pattern recognition receptors RIG-I and ZAP [15., 16., 17.], and it was recently identified as an RBP by proteome-wide approaches [9,18., 19., 20., 21.]. It has since been found that TRIM25’s E3 ubiquitin ligase activity is dependent on its interaction with RNA [22]. Recent methodological developments have enabled the proteome-wide discovery of novel RBDs present in unconventional RBPs (reviewed in [10]). For example, RBDmap combines protein–RNA crosslinking, partial proteolysis, and proteomics to identify the protein segments that engage with RNA on a global scale [19]. RBDmap revealed that, beyond classical RBDs, intrinsically disordered regions, enzymatic cores, and protein–protein interaction domains can be platforms for RNA binding.

In recent years, important efforts have been undertaken to elucidate the interactomes of specific RNA species. The different approaches taken to capture and characterise the protein–RNA interactions occurring on specific RNAs have been extensively reviewed [23]. In this review, we focus on how these methods have been adapted, and how others have been developed, to uncover the complement of proteins that interact with vRNA (i.e., the vRNA interactome).

## Approaches to uncover the viral RNA interactome

**Figure 1:**
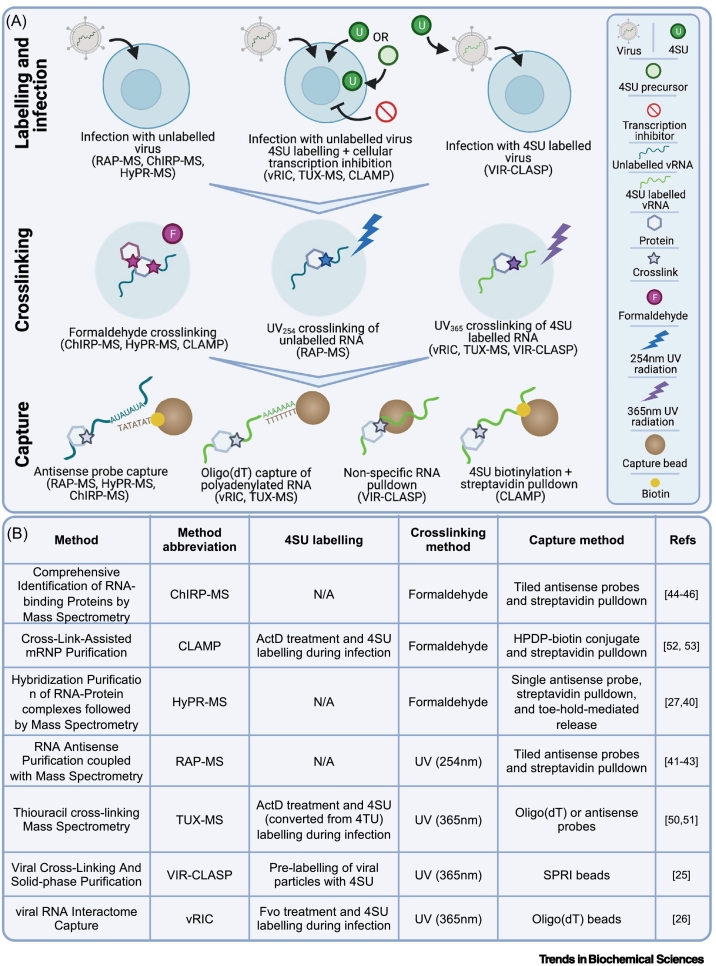
Approaches to elucidate the viral RNA (vRNA) interactome. (A) Schematic representation of the critical steps of the methods to elucidate vRNA–protein interactions and the alternatives for each step. The name and workflow of each approach are detailed in (B). Abbreviations: 4SU, 4-thiouridine; ActD, actinomycin D; ChIRP-MS, comprehensive identification of RNA-binding proteins by mass spectrometry; CLAMP, crosslink-assisted messenger ribonucleoprotein purification; Fvo, flavopiridol; HyPR-MS, hybridization purification of RNA–protein complexes followed by mass spectrometry; mRNP, messenger ribonucleoprotein; RAP-MS, RNA antisense purification and quantitative mass spectrometry; SPRI, solid-phase reversible immobilisation; TUX-MS, thiouracil cross-linking mass spectrometry; VIR-CLASP, viral cross-linking and solid-phase purification; vRIC, viral RNA interactome capture. See also [25., 26., 27.,40., 41., 42., 43., 44., 45.,50., 51., 52., 53.].

Although protein–vRNA interactions are fundamental for the viral life cycle, the complement of cellular RBPs that engage with vRNA remains poorly characterised. Over the past decade, several approaches have been developed to identify these critical host–virus interactions. These methods comprise four critical steps: (i) infection, (ii) protein–RNA crosslinking, (iii) RNA isolation, and (iv) proteomic analysis. Although these methods share similar workflows, each one approaches these steps in a distinct manner, as summarised in Figure 1. The success of these approaches relies on their ability to capture proteins that interact with vRNA while excluding those that do not (Box 1). Through understanding both the advantages and potential limitations of each method, it will be possible to evaluate their performance, interpret their results in light of case-specific experimental pitfalls, and choose the most appropriate workflow for a given experimental goal or system.

Box 1 **Identifying and controlling for sources of ‘noise’ in vRNA interactome studies**

Any vRNA interactome method will inevitably copurify contaminants or nonspecific/nonfunctional interactors (Figure I). It is therefore important that protocols (both experimental and analytical) are optimised to maximise signal-to-noise ratio and that appropriate controls are in place to account for sources of noise that cannot be eliminated. Here, we outline some such sources of noise (illustrated in Figure I) and the strategies that can be used to address them.

(A) Approaches that rely on formaldehyde crosslinking will isolate not only proteins binding directly with vRNA but also indirect protein interactors. It is thus possible that functionally unrelated long-distance interactors might be isolated due to protein–protein bridges or stabilisation of stochastic short-lived interactions. This can be mitigated by keeping formaldehyde concentration and incubation time to a minimum.
(B) All methods can copurify proteins binding noncovalently to target vRNA or its covalently bound proteins. To disrupt noncovalent interactions, stringent lysis and washing conditions with high-salt buffers, chaotropic detergents, and/or other denaturing agents should be used. A non-crosslinked control can help in determining the extent of noncovalent interactions following pulldown.
(C and D) Nontarget cellular RNAs can form partial hybrids with vRNA or probes during lysis and can be copurified due to the use of high–ionic strength buffers. If crosslinking is not limited to the target vRNA, contaminant RNAs will carry proteins covalently linked to them. Additional washes with low-salt buffers and a high-temperature pre-elution step can be included to disrupt nonspecific RNA–RNA interactions. Off-target interactions of probes can be accounted for with an uninfected control, and the nature and incidence of nontarget RNAs can be assessed by RNA sequencing.
(E) For methods that rely on 4SU labelling, it is important to minimise 4SU incorporation into nontarget RNAs. The dose of transcriptional inhibitor should be titrated to maximise inhibition of cellular transcription while minimising cytotoxicity. A mock-infected control incubated with 4SU and the inhibitor should be included to assess the extent of contamination derived from leaky transcription. If incorporation of 4SU into cellular RNAs is substantial, a specific vRNA capture method should be implemented.
(F) In CLAMP, capture relies on biotinylation of sulfhydryl groups in 4SU. Sulfhydryl groups are also present in other molecules in the cell, including cysteines, representing a source of contamination. This likely explains why there is a low incidence of proteins GO annotated as ‘RNA binding’ (~25%) in CLAMP studies compared with other methods (average of ~63%). RNase elution can reduce the incidence of these contaminants by eluting mostly RNA-associated proteins. Moreover, an uninfected control is critical to estimate the incidence of proteins that are isolated in a vRNA-independent manner.
(G) Proteins can bind directly to beads, probes, or resins. Stringent washing conditions and the inclusion of a non-crosslinked control are critical in reducing the incidence of contaminants. Choosing an elution method that only releases RNA-associated proteins, such as toehold or RNase elution, can also enhance methods’ specificity.

**Figure I:**
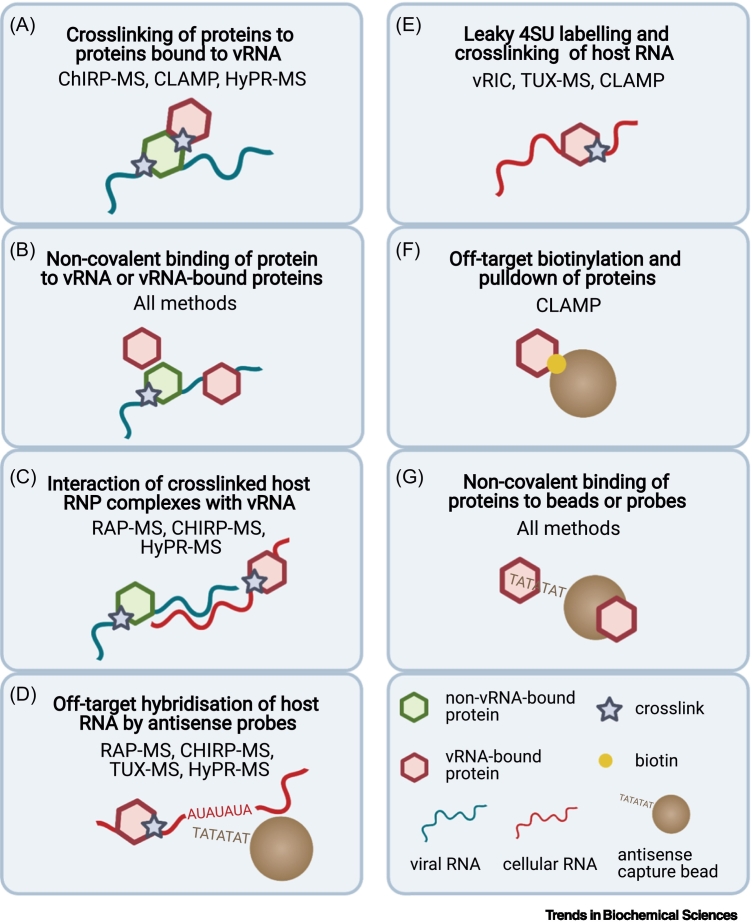
Sources of ‘noise’ in a viral (v)RNA interactome experiment. (A–G) Schematic representation of contaminants that may affect the different vRNA interactome approaches.

Alt-text: Box 1

### The infection model

The experimental design of a vRNA interactome experiment should consider the properties and life cycle of the virus under study. RNA viruses generate positive and negative sense RNAs to enable replication. In positive sense single-stranded (ss)RNA viruses, the genome also acts as an mRNA, and therefore its capture will reveal host factors involved in virtually all steps of the viral life cycle [3]. Conversely, in negative sense ssRNA viruses, the negative strand is the genome, and the positive strand acts as an mRNA [24]. Therefore, it is expected that positive and negative sense vRNAs may interact with a substantially different set of host proteins, and both should be captured to achieve a comprehensive vRNA interactome [25]. Moreover, other viruses generate more complex RNA expression patterns. For example, coronaviruses and alphaviruses produce subgenomic RNAs [20,26], whereas retroviruses produce alternatively spliced mRNAs encoding accessory proteins [27]. Given the diversity of vRNA species present in the infected cell, it is also of interest to generate species-specific vRNA interactomes. To this end, **antisense probes** can be designed that target features specific to particular vRNA species, such as splice sites. Such an approach was recently used to compare the interactomes of HIV type 1 (HIV-1) splice variants, identifying 212 proteins that potentially associate with specific HIV-1 RNAs [27].

Another important consideration is the time points after infection at which crosslinking and harvesting are performed. The choice of time points can be instrumental in enriching for host–virus interactions involved in particular steps of the viral life cycle. For example, proteins involved in replication and translation will be prominent early in infection, whereas viral particle assembly will grow in prevalence as infection progresses. Viral infection dynamics differ across viruses, so intracellular and extracellular vRNA, as well as viral protein abundance, should be measured to identify the most suitable time points [26] (Box 2). For these temporal analyses of the vRNA interactome, it is important to synchronise the infection so that cells become infected simultaneously. Synchronisation can be achieved by removing the virus-containing media after infection, followed by extensive washes and controlled trypsin treatment to inactivate remaining viral particles.

Box 2 **4SU incorporation approaches facilitate the study of the viral life cycle**

VIR-CLASP makes use of viral particles collected from cells cultured in 4SU-containing media and that thus have incorporated 4SU into their encapsidated vRNA [25]. Infection of cells in the absence of 4SU allows the study of the interactions that the incoming (4SU-labelled) vRNA establishes in the newly infected cell prior to the burst in viral replication. Although very valuable for studying early infection, one limitation is that this approach requires a very high **multiplicity of infection** (**MOI**) to maximise the proportion of 4SU-labelled vRNA molecules entering the cell. The use of high MOI is not amenable for all viruses and, in some cases, can alter the kinetics of infection [99].

Other methods, such as vRIC [26], TUX-MS [50,51], and CLAMP [52,53], combine 4SU RNA labelling with the use of an inhibitor of cellular transcription [actinomycin D (ActD) or flavopiridol (Fvo)] to study the interactions that vRNA establishes with the cell after the replication burst. Because the vRNA-dependent RNA polymerase (RDRP) is insensitive to these compounds, 4SU is mainly incorporated into nascent vRNAs. By altering the timing of inhibitor and 4SU addition, it might be possible to study specific stages in the viral life cycle and achieve a greater degree of temporal resolution. The success of these methods depends on maximising the efficiency of the transcriptional inhibition, 4SU vRNA labelling, and RNA isolation while minimising the labelling of nontarget RNAs and the toxic effects on the cell and the virus. It is therefore important to optimise the dose and incubation time of the transcription inhibitor and 4SU to minimise side effects. TUX-MS and CLAMP use ActD, which affects all cellular RNA polymerases (RNA pol). ActD-mediated inhibition allows exclusive incorporation of 4SU into vRNA but at the cost of substantial side effects on viral replication and cytotoxicity [100]. vRIC uses Fvo, which specifically inhibits RNA pol II and has no detectable effect on viral replication [26]. Because RNA pol I and III are not inhibited, Fvo must be combined with a downstream RNA isolation approach that does not isolate RNA transcribed by these polymerases, such as oligo(dT) or specific antisense probe capture.

Alt-text: Box 2

### ‘Freezing’ protein–RNA complexes with UV or chemical crosslinking

After infection of cultured cells, vRNA will undergo its replication cycle and will engage with cellular proteins. Once the infection has reached the desired stage, protein–vRNA interactions must be covalently immobilised to enable subsequent stringent purification of viral ribonucleoprotein (vRNP) complexes. The two most common protein–RNA crosslinking approaches rely on UV light and formaldehyde, both of which have benefits and drawbacks, as discussed in the following text.

UV irradiation of cells at 254 nm induces the formation of covalent bonds between RNA and proteins present at ‘zero’ distance (<2 Å) [28]. Because amino acid absorption is relatively low at 254 nm, UV crosslinking does not induce detectable levels of protein–protein crosslinking [19,28]. Although UV_254_ crosslinking has the benefit of being very selective, it suffers from low efficiency. Indeed, although its efficiency can reach nearly 40% for certain proteins, such as CELF1/CUGBP1, it averages 1–5% for most RBPs [9,29]. Proteins with high crosslinking efficiency are likely to establish long-lived interactions with RNA at sites with optimal nucleotide and amino acid composition and favourable geometry at the protein–RNA interface. UV crosslinking occurs preferentially at pyrimidines (especially uridines) and basic and aromatic residues [30]. Interactions between amino acids and nucleotide bases will also be favoured over those involving the ribose–phosphate backbone. This explains why UV crosslinking is more efficient for interactions with ssRNA than with dsRNA [9,10]. Another challenge for UV crosslinking is achieving high penetration in thick tissues, because UV will be absorbed at the sample’s surface. However, recent studies reported effective UV_254_ crosslinking in intact plant leaves by using repeated bursts of irradiation [31] and neuronal tissues through snap freezing and grinding prior to irradiation [14].

Another way to achieve UV crosslinking is by labelling RNA with **4-thiouridine** (**4SU**), a nucleotide analogue that is taken up by mammalian cells and is incorporated into nascent RNA when added to culture media [32]. 4SU has an additional absorbance peak spanning 312–370 nm and so promotes protein–RNA crosslinking upon irradiation with UV_312-370_, with UV_365_ being the most commonly used wavelength [9,33., 34., 35.]. Although initially reported to have higher efficiency than UV_254_ crosslinking, this is not evident when the two approaches are compared side by side. In general, the biases in amino acid and nucleotide preference are similar to UV_254_ crosslinking [30], although there are a few dozen RBPs favoured by one or another approach, reflecting differences in crosslinking chemistries [9]. The efficiency of crosslinking at UV_365_ is also heavily dependent on the level of incorporation of 4SU into the RNA. 4SU can be used to label *de novo* synthesised vRNAs and specifically study their interactomes, because natural bases exhibit negligible absorbance/crosslinking with UV_365_. Pulse labelling of vRNA with 4SU is a powerful approach to elucidate the vRNA interactome (Box 2) and can be paired with either nonspecific or specific RNA capture methods, increasing adaptability.

An alternative to UV crosslinking is the chemical crosslinker, formaldehyde, which has higher efficiency and tissue permeability than UV. Formaldehyde can form methylene bridges between amino/imino groups found across all proteins and nucleic acids, making it amenable for protein–protein, protein–DNA, and protein–RNA interaction studies [36]. Although all amino acids contain an amide group that can theoretically form crosslinks, most crosslinks occur at lysines due to the presence of the considerably more nucleophilic amino group in its side chain. Crosslinking also generally occurs between proteins/nucleic acids in close proximity as the methylene group has a span of ~2.3–2.7 Å [37].

The extent of formaldehyde crosslinking is highly dependent on the concentration and timing of treatment. When applied at high concentrations and over long periods of time (e.g., 4% for 30 min), it can stabilise organelles and large cellular macrostructures, making it a useful fixative for a broad range of applications, including microscopy. Even when lower concentrations and shorter crosslinking times are used, formaldehyde will inevitably induce the formation of protein–RNA, protein–DNA, and protein–protein bridges. In vRNA interactome studies, therefore, it will not only ‘freeze’ direct protein–RNA interactions but also protein–protein interactions formed by proteins bound to the vRNA [38,39], making formaldehyde crosslinking useful for studies where the experimental goal involves assessment of protein complexes instead of direct protein–RNA interactors.

### Isolation of protein–vRNA complexes

After ‘freezing’ protein–RNA interactions, vRNA and its covalently linked proteins are isolated. A common strategy to achieve this is to capture the vRNA specifically using antisense probes, which can be single probes [hybridization purification of RNA–protein complexes followed by **mass spectrometry (**HyPR-MS)] [27,40] or multiprobe sets [RNA antisense purification-quantitative MS (RAP-MS)] [41., 42., 43.], comprehensive identification of RNA-binding proteins by MS (ChIRP-MS) [44., 45., 46.]. Although multiprobe sets maximise vRNA recovery due to multiple simultaneous probe-target hybridisation events, each probe will have its own set of off-target interactions that will add to the overall experimental noise (Box 1). Conversely, single-probe approaches minimise noise at the cost of less efficient capture because they rely heavily on the accessibility of a single region of the vRNA. The inclusion of locked nucleotides into DNA probes [i.e., locked nucleic acids (LNAs)] can be used to improve the signal-to-noise ratio through maximising hybridisation strength and dsRNA invasion [47]. The specificity of the RNA capture can also be enhanced through the specific elution of target RNA (Box 1). For example, with LNA probes, it is possible to perform a first elution step at a relatively high temperature (~50°C) to remove contaminant RNAs that are captured via partial hybrids. This is followed by a second elution step at a higher temperature (70–90°C) that releases the target RNA captured through perfect base pairing [47., 48., 49.]. Alternatively, HyPR-MS uses toehold-mediated elution, where probe–RNA target interactions are displaced by a higher-affinity antisense probe [27,40].

An alternative to sequence-specific capture is to use bulk RNA purification in combination with vRNA-specific 4SU labelling and crosslinking (Box 1). **Oligo(dT) probes** enrich for polyadenylated [poly(A)] RNA and have been applied to study the RBP responses to severe acute respiratory syndrome coronavirus 2 (SARS-CoV-2) and Sindbis virus (SINV) infection [20,26]. Importantly, vRNA represents ~20% and ~70% of the RNA isolated by oligo(dT) in extracts from cells infected with SARS-CoV-2 [26] and SINV [20], respectively. This is because SARS-CoV-2 and SINV genomes and subgenomes have poly(A) tails and are very abundant in infected cells. Therefore, the combination of 4SU-labeled vRNA and oligo(dT) capture results in a high level of enrichment of vRNA-interacting proteins and has been successfully used by viral RNA interactome capture (vRIC) [26] and thiouracil cross-linking MS (TUX-MS) [50,51] (Figure 1 and Box 2).

Viral cross-linking and solid-phase purification (VIR-CLASP) also exploits 4SU labelling of vRNA (Box 2), but in combination with total RNA purification using solid-phase reversible immobilisation beads [25]. Alternatively, other recently developed total RNA isolation methods use organic phase separation [11,12,14] or silica columns [13] and can be applied to 4SU-labelling approaches, although this has not yet been applied in the context of viral infection. The benefit of total RNA capture methods is that they are not limited to polyadenylated vRNAs. However, this approach comes at a cost of copurifying a large proportion of non-vRNAs, with rRNA, small nucleolar (sno)RNA, tRNA, and small nuclear (sn)RNA being extremely abundant in eluates. The impact of these ‘contaminants’ on the quality of the vRNA interactome remains to be tested. Crosslink-assisted messenger RNP purification (CLAMP) combines formaldehyde crosslinking with another capture strategy, which consists of labelling vRNA with 4SU, followed by *in vitro* biotinylation of 4SU’s sulfhydryl group with an HPDP-biotin conjugate and purification of the biotinylated vRNA using streptavidin beads. Because biotinylation is performed in cellular extracts, biotin molecules can also bind to the sulfhydryl group present in the cysteine residues within proteins, likely contributing to high false-positive rates (Box 1) [52,53].

RNA tags, such as MS2 or tobramycin aptamers, have also been used extensively to study specific regions of viral genomes [54., 55., 56., 57.]. These ‘tags’ are RNA sequences that are known to form high-affinity interactions with a protein or small molecule, which can be used for specific affinity pulldown. The pitfall of these approaches is that vRNA must be genetically modified to incorporate the molecular tag, a process that is technically challenging and has the potential to disrupt viral replication. A limitation that applies specifically to the MS2 system is that it relies on an interaction between the MS2 bacteriophage coat protein and its target stem-loop structure derived from the bacteriophage’s RNA genome. This reliance on a native protein–RNA interaction makes it incompatible with the denaturing purification conditions required to remove nonspecific binders and contaminants [58].

### Proteomic analysis of vRNA interactomes

Once vRNP complexes have been isolated, proteins interacting with the vRNA are identified via MS. Although often overlooked, ensuring that the proteomics and the subsequent data analysis steps are robust is key to generating high-quality vRNA interactomes [59] (Box 3). Proteomic analysis of vRNA interactomes can be qualitative, where there is no comparison with a control group, or, as in the majority of cases, semiquantitative or quantitative, where a comparison of some type is performed. Semiquantitative analyses rely on present/absent comparisons between the infected sample and a control. Although present/absent comparisons are straightforward and quick to perform, the depth of comparison between samples is limited because any protein present in both samples is removed from the dataset. Quantitative analyses assign an abundance value to each protein in each sample, allowing enrichment analyses for all the identified proteins and increasing coverage [60]. However, appropriate statistical tests must be applied to assess the significance of the observed enrichments, because true hits are expected to be consistent across replicates [59]. Moreover, *p* values must be corrected using **false discovery rate** (**FDR**) estimation, which accounts for the false positives expected when testing the significance of multiple comparisons simultaneously.

Box 3 **Quantitative proteomic analyses applied to vRNA interactome studies**

Many of the studies discussed in this review rely on label-free quantification. In label-free applications, peptide quantitation is typically performed by analysing the signal (intensity) associated with the peptide or the number of sequencing events (spectral counts) for each peptide [58]. Although intensity and spectral count data are equally valid quantification approaches, it is important to ensure that spectral counts are handled using an appropriate processing method, such as that applied by SAINT [59]. Using intensity values offers the benefit of greater flexibility and depth in downstream analyses because intensity operates linearly over a much wider range of values than spectral counts.

One of the limitations of label-free analyses is that samples are analysed in separate mass spectrometer runs, which can be subject to additional technical sample-to-sample variability. Label-based quantitative approaches can be used to reduce technical variability. Stable isotope labelling allows multiple samples to be combined in a single run and can be performed during cell culture [stable isotope labelling by amino acids in cell culture (SILAC)] [60] or sample preparation (dimethyl labelling [61], TMT [64], iTRAQ [63]). Each label-based quantification approach has benefits and drawbacks. SILAC, for example, is compatible with any MS method at any depth and is very precise, but it is limited to only two- or three-way comparisons. Because the labelling is performed in the cultured cells, samples can be combined after lysis to reduce technical noise by performing the RNA capture in the combined sample [20,49]. The postelution labelling approaches TMT/iTRAQ allow multiplexing of up to 18 samples [64]. However, combining so many samples in the same MS run comes at a price of fewer peptide sequencing events per sample, which reduces the depth of the analysis. Fractionation is therefore recommended because it increases sequencing events per sample while maintaining the quantitative advantage of multiplexing [65]. Selecting the most appropriate label-free or label-dependent quantitative proteomic approach will depend on the experimental goal, the level of depth, and the need for multiplexing.

The performance of both label-free and label-based quantitative approaches is compromised if there is a lack of signal in one condition (‘zero value’), because these ‘zeros’ lead to ‘infinite’ or ‘zero’ ratios that cannot be analysed by statistical methods. There are semiquantitative approaches that deal with these scenarios by examining the distribution of ‘signal’ and ‘zeros’ across conditions and replicates. Conversely, imputation substitutes the ‘zeros’ for a value, which is often the ‘noise’ level in the experiment. Other imputation approaches account for other factors, such as intensity distribution, when calculating the ‘zero’ replacement value. Semiquantitative analyses provide a bidirectional (yes-or-no) answer that lacks quantitative information (amplitude of the effect). By contrast, imputation methods will generate protein intensity ratios between the two conditions at a cost of introducing potential artefacts. Despite drawbacks to either method, both semiquantitative and imputation approaches are particularly important for vRNA capture experiments because negative controls are often devoid of proteins, leading to a high incidence of ‘zero’ values.

Alt-text: Box 3

## Biological insights into viral infection from established vRNA interactome studies

**Figure 2:**
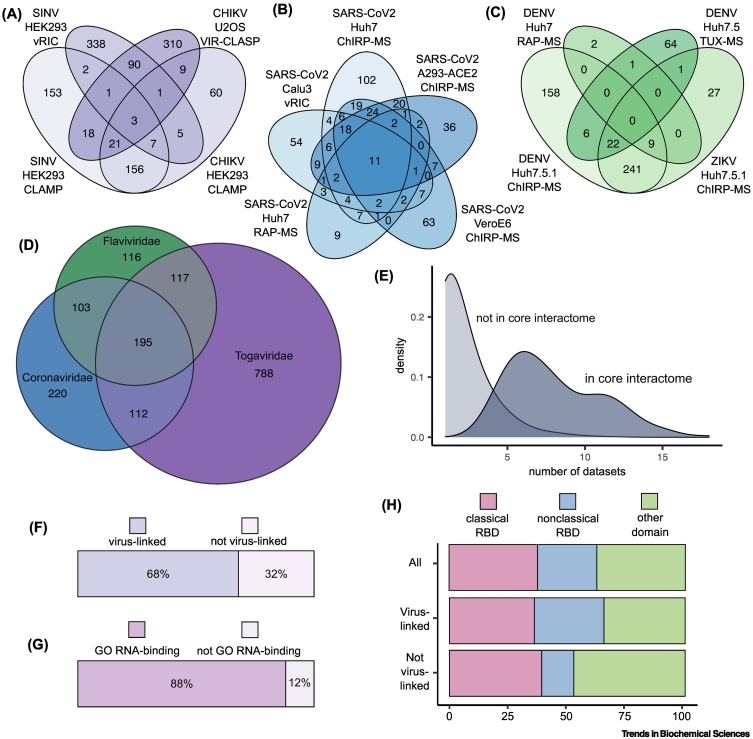
Defining the core viral RNA (vRNA) interactome. (A–C) Venn diagrams comparing the vRNA interactome datasets for the three viral families with multiple available datasets: (A) *Togaviridae* [25,54; W. Kamel, unpublished], (B) *Coronaviridae* [26,43,45,46], and (C) *Flaviviridae* [42,44,51]. Only datasets with at least two biological replicates were included. Cell type and capture method are listed under virus name. (D) Venn diagram comparing the *Togaviridae*, *Coronaviridae*, and *Flaviviridae* supersets. The overlap (195 proteins) between them is referred to here as the ‘core viral interactome.’ (E) Density plot showing the occurrence of proteins included and excluded in the core vRNA interactome in individual vRNA interactome datasets. (F) Proportion of the core vRNA interactome that has been linked to viral infection, either through gene ontology (GO) annotation or in the literature. PubMed was searched using the R package Rismed with the search terms [(protein) AND (virus OR viral)]. Any linked to viruses six or more times was classified as virus-linked. GO term annotation was extracted using the R package biomaRt. (G) Proportion of proteins in the vRNA interactome that are annotated with the GO term ‘RNA binding.’ (H) Proportion of proteins with classical RNA-binding domains (RBDs), nonclassical RBDs, and no known RBDs. This is shown for the core vRNA interactome, as well as its components that have been linked to infection and those without links (panel f). Individual protein-level information relating to panels f, g, and h is available in Supplementary Table 3. Abbreviations: CHIKV, Chikungunya virus; ChIRP-MS, comprehensive identification of RNA-binding proteins by mass spectrometry; CLAMP, crosslink-assisted messenger ribonucleoprotein purification; DENV, dengue virus; RAP-MS, RNA antisense purification and quantitative mass spectrometry; RBD, RNA-binding domain; SARS-CoV-2, severe acute respiratory syndrome coronavirus 2; SINV, Sindbis virus; TUX-MS, thiouracil cross-linking mass spectrometry; vRIC, viral RNA interactome capture; ZIKV, Zika virus.

The methods outlined above have thus far been used to generate 21 vRNA interactome datasets across 11 viruses from six viral families (Supplementary Table 1). When combined, these datasets comprise more than 2000 unique cellular proteins (Supplementary Table 2). Of these, 45% were identified in only a single study, highlighting the degree of heterogeneity across datasets. Indeed, when comparing datasets from viruses within the same family, the overlap is relatively poor (Figure 2A–C). Only 2.8%, 19.1%, and 5.8% of the RBPs were found in three or more of the *Togaviridae* (Figure 2A), *Coronaviridae* (Figure 2B), and *Flaviviridae* (Figure 2C) datasets, respectively.

The divergence between datasets is most likely caused by a combination of biological and technical factors, including the virus, cell line, experimental conditions, and method of choice. The influence of technical aspects is apparent in the fact that datasets generated using the same method applied to different viruses overlap to a greater extent than datasets for the same virus generated with different methods (Figure 2A–C). This variation might be introduced because of intrinsic limitations and biases of each vRNA interactome method or downstream at the proteomic data acquisition and analysis steps. The exact proteomic approach used and the depth of analysis achieved will have a major impact on the level of observed overlap. A study that lacks proteome depth will not detect low copy number proteins, leading to poor overlap with other datasets. Improving the depth and quality of proteomic analyses will therefore likely increase the overlap between high-quality datasets. It is also important to characterise the performance of each method through systematic validation of hits. A first level of validation would be to confirm vRNA–protein interactions via orthogonal strategies, such as crosslinking and immunoprecipitation (CLIP)–based and localisation approaches [20]. Moreover, the functional relevance for viral fitness of identified proteins should be comprehensively evaluated. Systematic assessment of dataset quality, along with identification of method-specific biases, should aid in identifying the most appropriate method for a given experimental goal. It could also allow the design of combinatorial approaches that take advantage of the strengths of multiple methods to provide a more holistic picture of the vRNA interactome.

Alongside this extensive heterogeneity, there are also important commonalities across viral families that may hint at conserved regulators of viral infection. Close to 200 proteins were present in vRNA interactomes from all three of the best-represented viral families (*Coronaviridae*, *Togaviridae*, and *Flaviviridae*), and we refer to these proteins here as the ‘core vRNA interactome’ (Figure 2D). Interestingly, proteins within the core vRNA interactome are identified, on average, in a higher number of datasets than proteins outside the core interactome (Figure 2E). This suggests that core vRNA interactome proteins interact with a wide range of vRNAs and/or are abundant enough in the eluates to be robustly identified, regardless of the method and the proteomic approach used. Moreover, 68% of the core interactome proteins have been previously linked to infection, either through **gene ontology** (**GO**) annotation or in peer-reviewed articles. It is important to stress that the other 32% lack established links to infection, representing potential unexplored avenues of research (Figure 2F).

The vast majority of the proteins within the core vRNA interactome are annotated by the GO term ‘RNA binding’ (Figure 2G), which is expected, given that these methods enrich for RBPs. Some of these proteins (~37%) contain classical RBDs, such as RNA recognition motifs (RRMs), K-homology domain (KH), or DEAD-box helicase domains [61]. These proteins are generally involved in canonical RNA processing pathways, such as translation and RNA splicing. Indeed, there are ten translation initiation and elongation factors in the core vRNA interactome, as well as 12 heterogeneous ribonucleoproteins (HNRNPs). HNRNPs have a modular architecture that often includes several RBDs [61] and serve a wide range of relatively well-characterised roles in RNA metabolism. HNRNPs have been extensively studied in the context of viruses and are ‘usual suspects’ in vRNA interactome analyses [1,2]. They establish long-lived and optimal interactions with RNA and often oligomerise, which likely leads to the crosslinking of multiple proteins to each RNA molecule. What is perhaps more exciting is that many RBPs display unconventional modes of RNA binding. For example, 25% of proteins have nonclassical RBDs, which are domains whose interaction with RNA has been experimentally proved but not characterised mechanistically. This is the case for the heat shock proteins HSP90AB1 and HSPA8 that harbour well-defined RNA-binding regions with unknown functional roles [1,19]. There are also 73 core vRNA interactome RBPs whose interactions with RNA have not yet been characterised. Interestingly, many of these unorthodox RBPs have not been linked to viral infection (Figure 2H), representing unexplored host–virus interactions.

**Figure 3:**
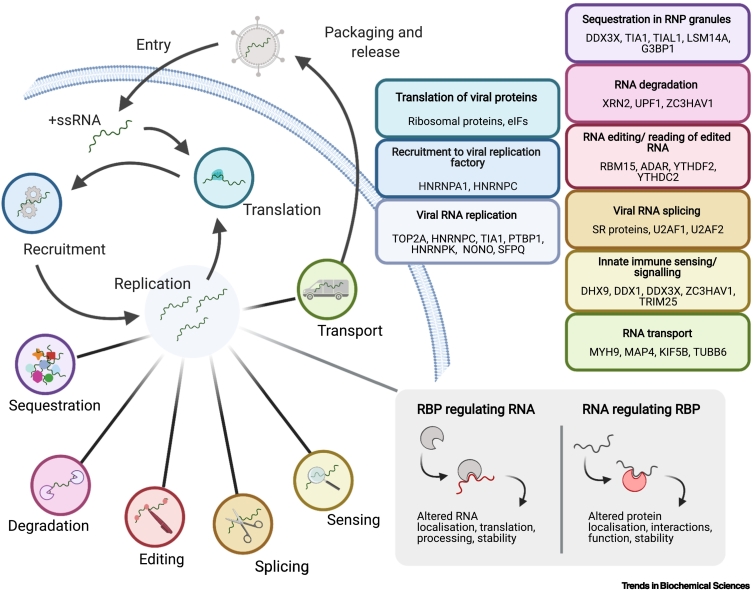
Cellular RNA-binding proteins (RBPs) participate in virtually all stages of viral infection. Schematic representation of the viral life cycle, highlighting the processes involved in viral (v)RNA metabolism. The members of the core vRNA interactome involved in these processes are highlighted in the coloured boxes. RBPs can regulate vRNA fate; however, emerging data suggest that vRNA may, in some instances, regulate protein function, as outlined in the grey box. This alternative regulatory process is referred to as ‘riboregulation.’ RNP, ribonucleoprotein; ssRNA, single-stranded RNA.

Many of the cellular RBPs within the core vRNA interactome have been shown to support or restrict virus infection, participating in key steps in the viral replication cycle, such as recruitment of vRNA to **viral replication factories**, vRNA replication, and translation [2,3] (Figure 3). In the following section, we describe some interesting examples of core vRNA interactome members that speak to important and emerging themes in vRNA biology.

### Innate immunity

Among the core vRNA interactome proteins, many have been linked to the cellular antiviral response. These include several helicases involved in vRNA sensing (DDX3X, DHX9, MOV10) and proteins involved in **stress granule** (**SG**) formation (G3BP1, TIA1). SGs are proposed to sequester vRNA to suppress viral replication and protein synthesis [62], so the presence of these proteins in the core vRNA interactome might be a reflection of the central role of this antiviral function in virus infection. These antiviral RBPs can be repurposed to support infection, and, indeed, the subgenomic RNA (sfRNA) of certain flaviviruses can sequester SG proteins to restrict SG formation [63]. This is an example of ‘**riboregulation**’ (Figure 3), which is an emerging concept in RNA biology that is likely to be an important part of many of the interactions taking place between vRNAs and host RBPs [10]. Riboregulation represents an inversion of the conventional paradigm that the role of RBPs is to regulate RNA function, instead positing that RNA is regulating RBP function. In recent years, an increasing number of examples of RNAs that regulate RBP function have been discovered, suggesting that this phenomenon is more broadly relevant than previously anticipated [10,64]. The idea that both protein and vRNA might be active players adds another level of complexity to understanding the roles of protein–RNA interactions in viral infection. For example, the enzymatic activities of the antiviral proteins TRIM25 [22] and protein kinase R (PKR) [65] are activated upon interaction with vRNA. These antiviral riboregulatory events can be disrupted by the virus. Indeed, HIV-1 has structural features within its genome that bind to PKR without causing activation, sequestering it to attenuate the antiviral response [66]. Although TRIM25 and PKR are powerful examples, we foresee that they represent the tip of the iceberg, and that there are dozens of riboregulatory events involving vRNA awaiting discovery.

### RNA stability and decay

Several proteins within the core vRNA interactome are involved in RNA stability and decay. These are well-characterised RBPs that harbour classical RBDs and include stability factors, such as PCBP2 and ELAVL1 [67], and antiviral factors, such as ZC3HAV1 (ZAP) [68], the nonsense-mediated decay factor UPF1 [69,70], and the exonuclease XRN2. XRN2 is a **5′-to-3′ exoribonuclease** involved in transcription termination, and, as a consequence, it exhibits mainly nuclear localisation [71,72]. However, a small cytoplasmic pool of this protein was shown to play an antiviral role in hepatitis C virus infection through inducing vRNA degradation [73]. Although its cytoplasmic counterpart, XRN1, has been better studied in the context of viral infection [20,74., 75., 76., 77.], the interplay between XRN2 and viruses remains largely unexplored. Interestingly, XRN1 is only present in one vRNA interactome dataset, whereas XRN2 is found in seven (Supplementary Table 3). These results highlight the importance of XRN2 in viral infection and call for further research to uncover its roles in the viral life cycle.

### Cytoskeleton-mediated transport

The cellular cytoskeleton is critical during infection in establishing appropriate localisation of viral particles, proteins, and vRNPs within cells [78]. The movement of vRNPs is thought to be promoted by the interaction between protein components of these complexes and **motor proteins**. However, dyneins, kinesins, and myosins have all been found to have RNA-binding activity [10,19]. The interaction of motor proteins with vRNA suggests a role for these protein–RNA interactions at regulating vRNP localisation. vRNA might act as a scaffold to recruit motor proteins and vRNP components into transport-active complexes. Alternatively, vRNA could play a more active role, such as through allosterically ‘riboregulating’ motor proteins [79].

MYH9 is a nonmuscle myosin present in the core vRNA interactome that has been linked to RNA transport [80]. It also plays an important role in cell-to-cell spread via **tunnelling nanotubes** (TNTs) in porcine reproductive and respiratory syndrome virus [81]– and infectious pancreatic necrosis virus [82]–infected cells. Several of the viruses whose RNA interacts with MYH9 are known to exploit TNTs for cell-to-cell spread [83., 84., 85.]. It will be interesting to establish whether vRNA is transported between cells in a capsid-free fashion and whether direct interaction between RNA and MYH9 might be involved in this process. Although a direct RNA-binding role for MYH9 has yet to be established, this protein is found in eight vRNA interactomes (Supplementary Table 3), using both UV and formaldehyde crosslinking, as well as in 17 cellular interactomes [86]. Furthermore, RBDmap revealed the presence of high-confidence RNA-binding surfaces at the myosin head of the MYH9 homologue, MYH14 [19]. The myosin head region of MYH14 has 80% homology with that of MYH9, and the RNA-bound peptide itself contains only conservative substitutions that would be unlikely to impair RNA-binding activity.

Although some proteins associated with the cytoskeleton promote motility, there are others whose roles are to inhibit it. Microtubule-associated protein 4 (MAP4) is involved in stabilising microtubule filaments and is known to be co-opted by some viruses, including human papillomavirus [87] and HIV-1 [88]. Although in these cases spindle stabilisation promotes viral replication, it can also restrict infection for viruses that rely on dynamic cytoskeleton networks. Depletion of MAP4 promotes influenza A virus (IAV) infection, suggesting that microtubule stabilisation impairs the transport of vRNPs to the nucleus [89]. Interestingly, MAP4 interacts with the RNA of eight viruses, including IAV [25] and HIV-1 [27,40], although the mechanism of this protein–RNA interaction in regulating infection remains to be elucidated. On the basis of RBDmap, MAP4 interacts with RNA via its tubulin-binding domain [19], raising the possibility that this interaction could allosterically regulate its microtubule-stabilising activity analogously to the inhibition of the autophagy factor p62 through its interaction with Vault 1-1 RNA (Figure 3) [10,22,64].

### DNA damage response

An unexpected set of proteins found recurrently in vRNA interactomes are those involved in **DNA damage response** (DDR). These proteins include the single-strand break repair protein PARP-1 and all three components of the DNA-dependent protein kinase (DNA-PK) complex (XRCC5, XRCC6, and PRKDC), which serves a crucial role in nonhomologous end joining [90]. The link between the DDR and viral infection has been extensively studied for DNA viruses [91] but remains poorly understood for RNA viruses [92]. DNA-PK has been linked to the immune response to DNA viruses as both ‘proviral’ [93] and antiviral [94] factors. It is plausible that DNA-PK mediates the DNA/RNA sensing role analogously to IFI16 [20,25,95,96]. The individual components of DNA-PK have been linked to various processes of RNA metabolism, including control of cellular transcription and translation [97,98]. Therefore, it is possible that DNA-PK components also contribute to vRNA metabolism. In agreement, XRCC5 depletion attenuates SARS-CoV-2 infection, hinting at a proviral role for this DNA-PK component [45]. In summary, the exact roles of DNA-PK and, more broadly, DDR in the life cycle of RNA viruses awaits better characterisation.

## Concluding remarks

The development of new proteome-wide methods to elucidate the composition of vRNPs has expanded our knowledge of the interactions that vRNA establishes with the host cell. These methods have been applied to 11 viruses and have both ‘rediscovered’ known regulators of infection and uncovered new host–virus interactions. However, our understanding of vRNP composition, function, and dynamics is still in its infancy. We envision that in coming years vRNP profiling approaches will be applied to (i) a broader range of viruses to shed light on compositional diversity; (ii) different times after infection to profile vRNP plasticity and dynamics throughout the virus life cycle; (iii) different cell types and hosts to explore the adaptability of vRNA interactions to different environmental conditions; and (iv) more physiological systems, such as primary cells, organoids, and tissues (see Outstanding questions). Moreover, we foresee a burst in our understanding of the molecular mechanisms underpinning the regulatory roles of cellular RBPs in virus infection. Together, global and mechanistic analyses of the vRNA interactome can lead to the discovery of new therapeutic targets, with potential for broad-spectrum antiviral activity.

Outstanding questions Technical divergence between viral ribonucleoprotein (vRNP) profiling methods often results in differences between datasets, even when these methods are applied to the same virus. How can we make use of the complementary strengths of each method to generate a near-complete viral (v)RNA interactome?

Dozens of RNA-binding proteins (RBPs) are consistently identified in the RNA interactomes of different viruses. Do these proteins represent panviral regulators of infection? How could we exploit these widespread host–virus interactions to generate broad-spectrum antivirals?

Current vRNA interactomes have been performed with non-synchronised infections and at time points at which most processes of the viral cycle are taking place simultaneously. Can we improve the temporal resolution of available methods to enrich for interactions at specific viral life cycle stages? Can we also increase specificity to isolate single vRNA species (e.g., genomic and subgenomic RNAs, as well as positive and negative sense strands)?

It is also critical to elucidate how cellular RBPs detect and bind to vRNA and exert their function to provide mechanistic insights into these host–virus interactions. Do cellular RBPs use known or novel RNA-binding domains (RBDs)? Do they recognise sequence and/or structural motifs? Are they subject to riboregulation or do they control vRNA fate?

While cell lines are a useful experimental tool, they do not fully recapitulate the complex network of host–virus interactions occurring *in vivo*. How can we adapt RBP studies to systems that are closer to a real infection, such as primary cells, organoids, tissues, or even whole organisms (e.g., mosquito)?

Alt-text: Outstanding questions

## Glossary

**4-Thiouridine (4SU)**: a photoreactive uridine analogue that is taken up by cells and incorporated into nascent RNA. 4SU-mediated protein–RNA crosslinking is induced by irradiation with UV light at 365 nm.
**5′****-to-3′ Exoribonuclease**: an enzyme that removes nucleotides from the 5′ end of RNA.
**Antisense probe**: a single-stranded (ss)DNA oligonucleotide with a sequence complementary to a region of the RNA of interest. They can vary in length (from 10 to ~100 nucleotides) and can be used singly or as a set, spanning the length of the vRNA. Probes can be hybridised to magnetic beads (generally through biotin–streptavidin interaction), which are used for pulldown.
**DNA damage response (DDR)**: a general term for the various processes involved in detecting and responding to DNA damage.
**False discovery rate (FDR)**: an adjusted *p* value that corrects for multiple testing when calculating the significance of hits in proteomic analysis.
**Gene ontology (GO)**: a formal representation of knowledge relating to the properties of gene products, focusing on three aspects: molecular function, biological process, and cellular component.
**Mass spectrometry (MS)**: an analytical approach for measuring the mass-to-charge ratio (*m*/*z*) of molecules present in a sample that can be applied for the high-throughput analysis of proteins (proteomics).
**Motor protein**: proteins able to move in an ATP-dependent manner along cytoskeletal networks and that are important for transport within cells.
**Multiplicity of infection (MOI)**: the number of viral particles per cell in an infection assay.
**Oligo(dT) probe**: an ssDNA oligonucleotide made of ~25 thymine nucleotides. This is complementary to the poly(A) tail found in cellular mRNAs and many vRNAs. The probes are hybridised to magnetic beads to facilitate pulldown.
**Riboregulation**: the process by which a protein’s function is altered through its interaction with RNA.
**RNA-binding domain (RBD)**: region of an RBP that engages in direct interaction with RNA. RBPs are often modular and harbour several RBDs to expand selectivity and affinity. Canonical RBDs include an RNA recognition motif, a K homology domain, and DEAD-box helicase, among others.
**RNA-binding protein (RBP)**: a protein that interacts with RNA. ‘Conventional’ RBPs interact with RNA through well-characterised RNA-binding domains (RBDs) and are generally involved in regulation of gene expression or RNA metabolism. ‘Unconventional’ RBPs use noncanonical modes of RNA binding such as RNA-binding surfaces in enzymatic cores, protein–protein interaction domains, or intrinsically disordered regions. They serve a wide range of functions, the full extent of which have yet to be elucidated.
**Stress granules (SGs)**: aggregates of protein and RNA that form in the cytoplasm of cells under stress and contain stalled replication complexes.
**Tunnelling nanotubes (TNTs)**: membranous bridges that can form between cells over relatively long distances, allowing intracellular communication and transport of cellular components.
**Viral replication factory**: an intracellular compartment formed during infection that houses the viral replication machinery and is the site of viral replication.
**Viral RNA (vRNA)**: a term to refer to all the RNA species generated by a virus during its life cycle. This includes both positive and negative strands, as well as subgenomic RNAs.
**Viral RNA interactome**: the full complement of proteins that interact with vRNA in a cell.

## Supplemental information

Supplementary tables Image 1
