# Supplementary material for: Uncovering viral RNA–host cell interactions on a proteome-wide scale
Source: Trends Biochem Sci. 2022 Jan;47(1):23–38. doi: 10.1016/j.tibs.2021.08.002 (PMC9187521; doi:10.1016/j.tibs.2021.08.002)
Supplement: Supplementary file 1 — Supplementary tables [file mmc1.docx]

**Supplemental information**

**Uncovering viral RNA-host cell interactions on a proteome-wide scale**

Louisa Iselin^1,2^, Natasha Palmalux^3^, Wael Kamel^2,3^, Peter Simmonds^1^, Shabaz Mohammed^2,4,5^, and Alfredo Castello*^2,3^

1. Nuffield Department of Medicine, Peter Medawar Building for Pathogen Research, University of Oxford, Oxford, OX13SY, UK
2. ﻿Department of Biochemistry, University of Oxford, South Parks Road, OX1 3QU, Oxford, UK.
3. ﻿MRC-University of Glasgow Centre for Virus Research, 464 Bearsden Road, Glasgow G61 1QH, Scotland (UK)
4. ﻿Department of Chemistry, Chemistry Research Laboratory, Mansfield Road, Oxford, OX1 3TA, UK
5. ﻿The Rosalind Franklin Institute, Oxfordshire, OX11 0FA, UK

﻿*Correspondence: [alfredo.castello@glasgow.ac.uk](mailto:alfredo.castello@glasgow.ac.uk)

**File inventory**

- **Supplemental Table 1.**
- **Supplemental Table 2.**
- **Supplemental Table 3.**

Supplementary table 1

| **Dataset** | **Ref** | **Virus** | **Family** | **Size** | **Timepoints** | **Cell type** | **Method** | **4SU labelling** | **Crosslinking method** | **Capture method** | **No. replicates** | **Proteomic analysis** | **Control for statistical analysis** | **Statistical analysis** |
| --- | --- | --- | --- | --- | --- | --- | --- | --- | --- | --- | --- | --- | --- | --- |
| Lee2020_SARSCoV2 | [41] | SARSCoV2 | *Coronaviridae* | 108 | 24 | Vero | RAP-MS | N/A | UV | Tiled antisense probes and streptavidin pulldown | 1 | Label-free quantification from spectral counts | Mock infection and no probe controls | Applied statistical test for spectra count enrichment, considering protein spectra count data as a binomial distribution to calculate P values. FDR of 5% was used to define hits. |
| Lee2020_OC43 | [41] | OC43 | *Coronaviridae* | 372 | 12, 24, 36, 48 | Vero | RAP-MS | N/A | UV | Tiled antisense probes and streptavidin pulldown | 1 | Label-free quantification from spectral counts | Mock infection and no probe controls | Applied statistical test for spectra count enrichment, considering protein spectra count data as a binomial distribution to calculate P values. FDR of 5% was used to define hits. |
| Kamel2020_SARSCoV2 | [26] | SARSCoV2 | *Coronaviridae* | 130 | 24 | Calu-3 | vRIC | Flavopiridol treatment and 4SU labelling during infection cycle | UV | Oligo(dT) beads | 4 | Label-free quantification from ion counts | Mock infection | Limma R package was used for analysis to perform empirical Bayesian method moderated t-test. FDR of 10% was used to define hits. |
| Schmidt2020_SARSCoV2 | [43] | SARSCoV2 | *Coronaviridae* | 48 | 24 | Huh7 | RAP-MS | N/A | UV | Tiled antisense probes and streptavidin pulldown | 2 | Quantitative analysis using TMT labelling | RMRP pulldown | Moderated two-sample t-test was used to compare enrichment in SARS-CoV2 RNA pulldown over RMRP pulldown. FDR of 5% was used to define hits. |
| Ooi2019_DENV | [44] | DENV | *Flaviviridae* | 436 | 48 | ﻿Huh7.5.1 | ChIRP-MS | N/A | Formaldehyde | Tiled antisense probes and streptavidin pulldown | 3 | Label-free quantification from spectral counts | Mock infection | SAINT scoring system (SAINTq) was used to assess significance. FDR ﻿< 1%, SAINT score >0.99 and enrichment >2 fold over the uninfected control were used to define hits. |
| Ooi2019_ZIKV | [44] | ZIKV | *Flaviviridae* | 300 | 48 | ﻿Huh7.5.1 | ChIRP-MS | N/A | Formaldehyde | Tiled antisense probes and streptavidin pulldown | 3 | Label-free quantification from spectral counts | Mock infection | SAINT scoring system (SAINTq) was used to assess significance. FDR ﻿< 1%, SAINT score >0.99 and enrich- ment >2 fold over the uninfected control were used to define hits. |
| Ooi2019_RV | [44] | RV | *Picornaviridae* | 340 | 48 | HeLa | ChIRP-MS | N/A | Formaldehyde | Tiled antisense probes and streptavidin pulldown | 1 | Label-free quantification from spectral counts | Mock infection | SAINT scoring system (SAINTq) was used to assess significance. FDR ﻿< 1%, SAINT score >0.99 and enrichment >2 fold over the uninfected control were used to define hits. |
| Gebhart2020_SINV | [53] | SINV | *Togaviridae* | 361 | 6 | HEK293 | CLAMP | Actinomycin D treatment and 4SU labelling during infection cycle | Formaldehyde | HPDP-biotin conjugate and streptavidin pulldown | 2 | Qualitative analysis | Mock infection | Hits were defined as proteins present in the infected sample but absent from the uninfected control. No statistical cut-off was used. |
| Gebhart2020_CHIKV | [53] | CHIKV | *Togaviridae* | 262 | 6 | HEK293 | CLAMP | Actinomycin D treatment and 4SU labelling during infection cycle | Formaldehyde | HPDP-biotin conjugate and streptavidin pulldown | 2 | Qualitative analysis | Mock infection | Hits were defined as proteins present in the infected sample but absent from the uninfected control. No statistical cut-off was used. |
| Gebhart2020_VEE | [53] | VEE | *Togaviridae* | 209 | 6 | HEK293 | CLAMP | Actinomycin D treatment and 4SU labelling during infection cycle | Formaldehyde | HPDP-biotin conjugate and streptavidin pulldown | 2 | Qualitative analysis | Mock infection | Hits were defined as proteins present in the infected sample but absent from the uninfected control. No statistical cut-off was used. |
| Lenarcic2013_PV | [50] | PV | *Picornaviridae* | 82 | 5 | HeLa | TUX-MS | Actinomycin D treatment and 4SU labelling during infection cycle (4TU is added and converted to 4SU by endogenously expressed UPRT | UV | Oligo(dT) beads | 1 | Label-free quantification from spectral counts | Mock infection | Hits determined based on >2-fold enrichment over mock infected cells. No statistical cut-off was used. |
| Phillips2016_DENV | [42] | DENV | *Flaviviridae* | 12 | 30 | Huh7 | RAP-MS | N/A | UV | Tiled antisense probes and streptavidin pulldown | 3 | Label-free quantification from spectral counts | Mock infection | Hits determined based on >2-fold enrichment over mock infected cells in at least 2 of the 3 replicates. No statistical cut-off was used. |
| Viktorovskaya2016_DENV | [51] | DENV | *Flaviviridae* | 94 | 48 | Huh7.5 | TUX-MS | Actinomycin D treatment and 4SU labelling during infection cycle (4TU is added and converted to 4SU by endogenously expressed UPRT | UV | Tiled antisense probes and streptavidin pulldown | 2 | Quantitative analysis using SILAC labelling | Mock infection | Hits were determined based on >1.5-fold enrichment over mock infected cells. No statistical cut-off was used. |
| Koener2017_HIV | [40] | HIV | *Retroviridae* | 188 | 48 | Jurkat | HyPR-MS | N/A | Formaldehyde | Single antisense probe, streptavidin pulldown, and toe-hold-mediated release | 3 | Label-free quantification from ion counts | Scrambled probe | Student's T-test was performed, comparing HIV-specific capture to scrambled control probe capture. Student's T-test test statistic was calculated with ﻿S0 = 0.8. Hits were determined as proteins that passed a permutation-based 1% FDR threshold. These had maximum p-value of 0.05 and minimum fold-change of 2.2. |
| Kim2020_IAV | [25] | IAV | *Orthomyxoviridae* | 317 | 1 | A549 | VIR-CLASP | Pre-labelling of viral particles with 4SU | UV | SPRI beads | 2 | Label-free quantification from ion counts | Unlabelled virus infection  (no 4SU) | ﻿Moderated T test was calculated using limma R package. Hit were determined based on FDR < 1% and a fold change >5 over cells infected with unlabelled virus. |
| Knoener2021_HIV | [27] | HIV | *Retroviridae* | 926 | 48 | Jurkat | HyPR-MS | N/A | Formaldehyde | Repeated antisense probe captures, streptavidin pulldown, and toe-hold-mediated release | 3 | Label-free quantification from ion counts | None | Comparisons between splice variants were performed but, because there was no mock, no statistical or fold-change cut-off was used to define hits. |
| Kim2020_CHIKV | [25] | CHIKV | *Togaviridae* | 453 | 0.2, 1, 3 | ﻿U2OS | VIR-CLASP | Pre-labelling of viral particles with 4SU | UV | SPRI beads | 3 | Label-free quantification from ion counts | Unlabelled virus infection  (no 4SU) | ﻿Moderated T test was calculated using limma R package. Hit were determined based on FDR < 1% and a fold change >5 over cells infected with unlabelled virus. |
| Kamel2021_SINV | NA | SINV | *Togaviridae* | 227 | 24 | HEK293 | vRIC | Flavopiridol treatment and 4SU labelling during infection cycle | UV | Oligo(dT) beads | 4 | Label-free quantification from ion counts | Mock infection | Limma R package was used for analysis to perform empirical Bayesian method moderated t-test. FDR of 5% and >2-fold enrichment over uninfected control were used to define hits. |
| Flynn2020h_SARSCoV2 | [46] | SARSCoV2 | *Coronaviridae* | 229 | 24, 48 | Huh7.5 | ChIRP-MS | N/A | Formaldehyde | Tiled antisense probes and streptavidin pulldown | 3 | Label-free quantification from ion counts | Mock infection | ﻿P values were calculated using DEP default workflow. Hits were determined as proteins with FDR ≤ 5% and fold change > 0 of mock infected cells. |
| Flynn2020v_SARSCoV2 | [46] | SARSCoV2 | *Coronaviridae* | 163 | 24, 48 | VeroE6 | ChIRP-MS | N/A | Formaldehyde | Tiled antisense probes and streptavidin pulldown | 3 | Label-free quantification from ion counts | Mock infection | ﻿P values were calculated using DEP default workflow. Hits were determined as proteins with FDR ≤ 5% and fold change > 0 of mock infected cells. |
| Labeau2021_SARSCoV2 | [45] | SARSCoV2 | *Coronaviridae* | 140 | 48 | 293T-ACE2 | ChIRP-MS | N/A | Formaldehyde | Tiled antisense probes and streptavidin pulldown | 5 | Label-free quantification from spectral counts | Mock infection | ﻿SAINTexpress was used for comparison with mock. ﻿Hits were considered those with MIST scoring > 0.7, and a SAINTexpress score above 0.78. |

Supplementary Table 2

| protein | count | Flynn  2020hSARSCoV2 | Flynn  2020v  SARS  CoV2 | Kim  2020  CHIKV | Kim  2020  IAV | Lee  2020  OC43 | Lee  2020  SARS  CoV2 | Kamel  2020  SARS  CoV2 | Schmidt  2020  SARS  CoV2 | Ooi  2019  DENV | Ooi  2019  ZIKV | Ooi  2019  RV | Gebhart  2020  SINV | Gebhart  2020  CHIKV | Gebhart2020  VEEV | Lenarcic  2013  PV | Phillips  2016  DENV | Viktorovskaya  2016  DENV | Koener  2017  HIV | Knoener  2021  HIV | Kamel  2021  SINV | Labeau  2021  SARS  CoV2 |
| --- | --- | --- | --- | --- | --- | --- | --- | --- | --- | --- | --- | --- | --- | --- | --- | --- | --- | --- | --- | --- | --- | --- |
| FAM120A | 12 | TRUE | TRUE | FALSE | FALSE | TRUE | TRUE | TRUE | FALSE | TRUE | TRUE | TRUE | FALSE | FALSE | FALSE | FALSE | FALSE | FALSE | TRUE | TRUE | TRUE | TRUE |
| FUBP1 | 12 | TRUE | TRUE | FALSE | FALSE | TRUE | FALSE | FALSE | FALSE | TRUE | TRUE | TRUE | FALSE | FALSE | TRUE | FALSE | FALSE | TRUE | TRUE | TRUE | TRUE | TRUE |
| HDLBP | 14 | TRUE | TRUE | TRUE | TRUE | TRUE | TRUE | TRUE | TRUE | TRUE | TRUE | TRUE | FALSE | FALSE | FALSE | FALSE | FALSE | FALSE | FALSE | TRUE | TRUE | TRUE |
| HNRNPA1 | 15 | TRUE | TRUE | TRUE | FALSE | TRUE | FALSE | TRUE | TRUE | TRUE | TRUE | TRUE | TRUE | TRUE | TRUE | FALSE | FALSE | FALSE | TRUE | TRUE | FALSE | TRUE |
| HNRNPA2B1 | 13 | TRUE | FALSE | TRUE | FALSE | TRUE | FALSE | TRUE | FALSE | TRUE | TRUE | TRUE | TRUE | TRUE | TRUE | FALSE | TRUE | FALSE | FALSE | TRUE | FALSE | TRUE |
| HNRNPA3 | 11 | TRUE | FALSE | TRUE | FALSE | TRUE | TRUE | TRUE | FALSE | TRUE | TRUE | TRUE | FALSE | FALSE | FALSE | TRUE | FALSE | FALSE | FALSE | TRUE | FALSE | TRUE |
| HNRNPAB | 10 | TRUE | FALSE | FALSE | FALSE | TRUE | TRUE | FALSE | FALSE | TRUE | TRUE | TRUE | TRUE | TRUE | TRUE | FALSE | FALSE | FALSE | FALSE | TRUE | FALSE | FALSE |
| HNRNPC | 13 | TRUE | TRUE | FALSE | FALSE | TRUE | FALSE | TRUE | FALSE | TRUE | TRUE | TRUE | TRUE | FALSE | FALSE | FALSE | TRUE | FALSE | TRUE | TRUE | TRUE | TRUE |
| HNRNPH1 | 9 | TRUE | TRUE | FALSE | FALSE | TRUE | FALSE | FALSE | FALSE | TRUE | TRUE | TRUE | TRUE | FALSE | FALSE | FALSE | FALSE | FALSE | FALSE | TRUE | FALSE | TRUE |
| HNRNPK | 14 | TRUE | FALSE | TRUE | TRUE | TRUE | FALSE | FALSE | FALSE | TRUE | TRUE | TRUE | TRUE | TRUE | TRUE | TRUE | FALSE | FALSE | TRUE | TRUE | FALSE | TRUE |
| HNRNPL | 13 | TRUE | TRUE | TRUE | FALSE | TRUE | TRUE | TRUE | FALSE | TRUE | TRUE | TRUE | FALSE | FALSE | FALSE | TRUE | FALSE | TRUE | FALSE | TRUE | FALSE | TRUE |
| HNRNPM | 14 | TRUE | TRUE | FALSE | FALSE | TRUE | TRUE | TRUE | FALSE | TRUE | TRUE | TRUE | TRUE | TRUE | FALSE | FALSE | FALSE | TRUE | FALSE | TRUE | TRUE | TRUE |
| IGF2BP1 | 9 | TRUE | FALSE | FALSE | FALSE | FALSE | FALSE | TRUE | FALSE | TRUE | TRUE | TRUE | FALSE | FALSE | FALSE | FALSE | FALSE | FALSE | TRUE | TRUE | TRUE | TRUE |
| IGF2BP3 | 11 | TRUE | TRUE | FALSE | FALSE | TRUE | TRUE | TRUE | FALSE | TRUE | TRUE | TRUE | FALSE | FALSE | FALSE | FALSE | FALSE | FALSE | FALSE | TRUE | TRUE | TRUE |
| L1RE1 | 5 | TRUE | FALSE | FALSE | FALSE | FALSE | FALSE | TRUE | FALSE | TRUE | TRUE | FALSE | FALSE | FALSE | FALSE | FALSE | FALSE | FALSE | FALSE | FALSE | TRUE | FALSE |
| MATR3 | 11 | TRUE | FALSE | FALSE | TRUE | TRUE | TRUE | FALSE | FALSE | TRUE | TRUE | TRUE | FALSE | FALSE | FALSE | FALSE | FALSE | TRUE | FALSE | TRUE | TRUE | TRUE |
| PABPC1 | 13 | TRUE | TRUE | FALSE | FALSE | TRUE | TRUE | TRUE | TRUE | TRUE | TRUE | TRUE | FALSE | FALSE | FALSE | FALSE | TRUE | FALSE | FALSE | TRUE | TRUE | TRUE |
| PTBP1 | 13 | TRUE | TRUE | TRUE | FALSE | TRUE | TRUE | TRUE | FALSE | TRUE | TRUE | TRUE | TRUE | FALSE | FALSE | FALSE | FALSE | TRUE | FALSE | TRUE | FALSE | TRUE |
| PURA | 10 | TRUE | TRUE | FALSE | FALSE | TRUE | TRUE | TRUE | FALSE | TRUE | TRUE | FALSE | FALSE | FALSE | FALSE | TRUE | FALSE | FALSE | FALSE | FALSE | TRUE | TRUE |
| RACK1 | 6 | TRUE | FALSE | TRUE | FALSE | FALSE | FALSE | FALSE | FALSE | TRUE | TRUE | TRUE | FALSE | FALSE | FALSE | FALSE | FALSE | FALSE | FALSE | FALSE | FALSE | TRUE |
| RPL15 | 8 | TRUE | FALSE | TRUE | TRUE | FALSE | FALSE | FALSE | TRUE | TRUE | TRUE | FALSE | FALSE | FALSE | FALSE | FALSE | FALSE | FALSE | FALSE | TRUE | TRUE | FALSE |
| RPL24 | 7 | TRUE | FALSE | FALSE | FALSE | TRUE | TRUE | FALSE | FALSE | TRUE | FALSE | TRUE | FALSE | FALSE | FALSE | FALSE | FALSE | FALSE | FALSE | TRUE | TRUE | FALSE |
| RPS14 | 8 | TRUE | FALSE | FALSE | FALSE | TRUE | TRUE | FALSE | TRUE | TRUE | TRUE | FALSE | TRUE | FALSE | FALSE | FALSE | FALSE | FALSE | FALSE | TRUE | FALSE | FALSE |
| RRBP1 | 9 | TRUE | TRUE | TRUE | TRUE | TRUE | FALSE | FALSE | FALSE | TRUE | TRUE | FALSE | FALSE | FALSE | FALSE | FALSE | FALSE | FALSE | FALSE | TRUE | FALSE | TRUE |
| SFPQ | 16 | TRUE | TRUE | FALSE | TRUE | TRUE | TRUE | FALSE | FALSE | TRUE | TRUE | TRUE | TRUE | TRUE | TRUE | TRUE | FALSE | TRUE | FALSE | TRUE | TRUE | TRUE |
| SRSF2 | 7 | TRUE | TRUE | FALSE | FALSE | FALSE | FALSE | FALSE | FALSE | TRUE | TRUE | TRUE | TRUE | FALSE | FALSE | FALSE | FALSE | FALSE | FALSE | TRUE | FALSE | FALSE |
| SRSF6 | 8 | TRUE | TRUE | TRUE | FALSE | TRUE | FALSE | FALSE | FALSE | TRUE | TRUE | TRUE | FALSE | FALSE | FALSE | FALSE | FALSE | FALSE | FALSE | TRUE | FALSE | FALSE |
| TARDBP | 7 | TRUE | FALSE | TRUE | FALSE | TRUE | FALSE | FALSE | FALSE | TRUE | TRUE | TRUE | FALSE | FALSE | FALSE | FALSE | FALSE | FALSE | FALSE | FALSE | FALSE | TRUE |
| TIA1 | 9 | TRUE | TRUE | FALSE | FALSE | TRUE | FALSE | TRUE | FALSE | TRUE | FALSE | TRUE | FALSE | FALSE | FALSE | TRUE | FALSE | FALSE | FALSE | FALSE | TRUE | TRUE |
| YBX1 | 15 | TRUE | FALSE | FALSE | FALSE | TRUE | TRUE | TRUE | TRUE | TRUE | TRUE | TRUE | FALSE | TRUE | FALSE | TRUE | TRUE | FALSE | TRUE | TRUE | TRUE | TRUE |
| ZCCHC3 | 4 | TRUE | FALSE | FALSE | FALSE | FALSE | FALSE | FALSE | FALSE | TRUE | TRUE | FALSE | FALSE | FALSE | FALSE | FALSE | FALSE | FALSE | FALSE | FALSE | TRUE | FALSE |
| ZNF638 | 8 | TRUE | TRUE | FALSE | TRUE | FALSE | FALSE | FALSE | FALSE | TRUE | TRUE | TRUE | FALSE | FALSE | FALSE | TRUE | FALSE | FALSE | FALSE | FALSE | TRUE | FALSE |
| ACLY | 8 | TRUE | FALSE | TRUE | FALSE | FALSE | FALSE | FALSE | FALSE | TRUE | TRUE | TRUE | TRUE | TRUE | FALSE | FALSE | FALSE | FALSE | FALSE | TRUE | FALSE | FALSE |
| AHCY | 8 | TRUE | FALSE | FALSE | FALSE | FALSE | FALSE | FALSE | FALSE | TRUE | TRUE | TRUE | TRUE | TRUE | FALSE | FALSE | FALSE | FALSE | TRUE | TRUE | FALSE | FALSE |
| ATIC | 6 | TRUE | FALSE | TRUE | FALSE | FALSE | FALSE | FALSE | FALSE | TRUE | TRUE | FALSE | FALSE | FALSE | FALSE | FALSE | FALSE | FALSE | FALSE | TRUE | TRUE | FALSE |
| C1QBP | 5 | TRUE | FALSE | FALSE | FALSE | FALSE | FALSE | FALSE | FALSE | FALSE | FALSE | FALSE | TRUE | TRUE | TRUE | FALSE | FALSE | TRUE | FALSE | FALSE | FALSE | FALSE |
| CNBP | 10 | TRUE | TRUE | FALSE | FALSE | TRUE | TRUE | TRUE | TRUE | FALSE | FALSE | FALSE | FALSE | TRUE | TRUE | TRUE | TRUE | FALSE | FALSE | FALSE | FALSE | FALSE |
| CNOT1 | 5 | TRUE | FALSE | TRUE | TRUE | FALSE | FALSE | FALSE | FALSE | TRUE | TRUE | FALSE | FALSE | FALSE | FALSE | FALSE | FALSE | FALSE | FALSE | FALSE | FALSE | FALSE |
| COPB2 | 5 | TRUE | FALSE | TRUE | FALSE | FALSE | FALSE | FALSE | FALSE | TRUE | TRUE | FALSE | FALSE | FALSE | FALSE | FALSE | FALSE | FALSE | FALSE | TRUE | FALSE | FALSE |
| CSDE1 | 11 | TRUE | FALSE | FALSE | FALSE | TRUE | TRUE | TRUE | TRUE | TRUE | TRUE | TRUE | FALSE | FALSE | FALSE | FALSE | TRUE | FALSE | FALSE | TRUE | TRUE | FALSE |
| DDX3X | 12 | TRUE | FALSE | TRUE | TRUE | TRUE | TRUE | TRUE | TRUE | TRUE | TRUE | TRUE | FALSE | FALSE | FALSE | FALSE | FALSE | FALSE | FALSE | TRUE | TRUE | FALSE |
| DHX9 | 13 | TRUE | TRUE | TRUE | TRUE | TRUE | FALSE | FALSE | FALSE | TRUE | TRUE | TRUE | FALSE | TRUE | FALSE | TRUE | FALSE | TRUE | FALSE | TRUE | FALSE | TRUE |
| EIF2S1 | 6 | TRUE | TRUE | FALSE | FALSE | FALSE | FALSE | FALSE | FALSE | TRUE | TRUE | FALSE | TRUE | FALSE | FALSE | FALSE | FALSE | FALSE | FALSE | TRUE | FALSE | FALSE |
| EIF4A1 | 11 | TRUE | TRUE | FALSE | TRUE | TRUE | FALSE | TRUE | FALSE | TRUE | TRUE | TRUE | FALSE | FALSE | FALSE | TRUE | FALSE | FALSE | FALSE | TRUE | TRUE | FALSE |
| EIF4B | 11 | TRUE | FALSE | FALSE | FALSE | TRUE | TRUE | TRUE | TRUE | TRUE | TRUE | TRUE | FALSE | FALSE | FALSE | TRUE | TRUE | FALSE | FALSE | FALSE | TRUE | FALSE |
| EIF4H | 15 | TRUE | TRUE | FALSE | FALSE | TRUE | TRUE | TRUE | TRUE | TRUE | TRUE | TRUE | TRUE | TRUE | TRUE | TRUE | FALSE | FALSE | FALSE | TRUE | FALSE | TRUE |
| EZR | 5 | TRUE | FALSE | TRUE | FALSE | FALSE | FALSE | FALSE | FALSE | TRUE | TRUE | TRUE | FALSE | FALSE | FALSE | FALSE | FALSE | FALSE | FALSE | FALSE | FALSE | FALSE |
| FEN1 | 5 | TRUE | FALSE | FALSE | FALSE | FALSE | FALSE | FALSE | FALSE | TRUE | FALSE | TRUE | FALSE | FALSE | FALSE | FALSE | FALSE | FALSE | FALSE | TRUE | TRUE | FALSE |
| FUBP3 | 11 | TRUE | TRUE | FALSE | FALSE | TRUE | TRUE | TRUE | FALSE | TRUE | TRUE | TRUE | FALSE | FALSE | FALSE | FALSE | FALSE | FALSE | FALSE | TRUE | TRUE | TRUE |
| FXR1 | 12 | TRUE | TRUE | FALSE | FALSE | TRUE | TRUE | TRUE | FALSE | TRUE | TRUE | TRUE | FALSE | FALSE | FALSE | TRUE | FALSE | FALSE | TRUE | TRUE | TRUE | FALSE |
| G3BP1 | 11 | TRUE | TRUE | FALSE | FALSE | TRUE | TRUE | TRUE | FALSE | TRUE | TRUE | TRUE | FALSE | FALSE | FALSE | FALSE | FALSE | FALSE | FALSE | TRUE | TRUE | TRUE |
| GDI2 | 6 | TRUE | FALSE | TRUE | FALSE | FALSE | FALSE | FALSE | FALSE | TRUE | TRUE | TRUE | FALSE | FALSE | FALSE | FALSE | FALSE | FALSE | FALSE | TRUE | FALSE | FALSE |
| HNRNPD | 13 | TRUE | TRUE | FALSE | FALSE | TRUE | TRUE | FALSE | FALSE | TRUE | TRUE | TRUE | TRUE | FALSE | TRUE | TRUE | FALSE | FALSE | TRUE | TRUE | FALSE | TRUE |
| HNRNPDL | 11 | TRUE | TRUE | FALSE | FALSE | TRUE | FALSE | TRUE | FALSE | TRUE | TRUE | TRUE | TRUE | FALSE | TRUE | FALSE | FALSE | FALSE | FALSE | TRUE | FALSE | TRUE |
| HNRNPU | 11 | TRUE | TRUE | TRUE | TRUE | TRUE | FALSE | FALSE | FALSE | TRUE | FALSE | TRUE | TRUE | FALSE | FALSE | TRUE | FALSE | FALSE | FALSE | TRUE | FALSE | TRUE |
| HSPA4 | 7 | TRUE | FALSE | FALSE | TRUE | FALSE | FALSE | FALSE | FALSE | TRUE | FALSE | TRUE | TRUE | FALSE | FALSE | FALSE | FALSE | FALSE | FALSE | TRUE | FALSE | TRUE |
| IGF2BP2 | 10 | TRUE | TRUE | FALSE | FALSE | TRUE | TRUE | TRUE | TRUE | TRUE | TRUE | FALSE | FALSE | FALSE | FALSE | FALSE | FALSE | FALSE | FALSE | FALSE | TRUE | TRUE |
| ILF3 | 13 | TRUE | TRUE | TRUE | TRUE | TRUE | TRUE | FALSE | FALSE | TRUE | TRUE | TRUE | FALSE | FALSE | FALSE | TRUE | FALSE | TRUE | FALSE | TRUE | FALSE | TRUE |
| MAP4 | 12 | TRUE | FALSE | TRUE | TRUE | TRUE | TRUE | FALSE | FALSE | TRUE | TRUE | FALSE | FALSE | TRUE | TRUE | FALSE | FALSE | FALSE | TRUE | TRUE | FALSE | TRUE |
| MOV10 | 12 | TRUE | TRUE | TRUE | FALSE | TRUE | TRUE | FALSE | TRUE | TRUE | TRUE | TRUE | FALSE | FALSE | FALSE | FALSE | FALSE | FALSE | TRUE | TRUE | FALSE | TRUE |
| NCL | 18 | TRUE | TRUE | TRUE | TRUE | TRUE | FALSE | TRUE | FALSE | TRUE | TRUE | TRUE | TRUE | TRUE | TRUE | TRUE | TRUE | FALSE | TRUE | TRUE | TRUE | TRUE |
| PABPC4 | 13 | TRUE | TRUE | TRUE | FALSE | TRUE | TRUE | TRUE | TRUE | TRUE | TRUE | TRUE | FALSE | FALSE | FALSE | FALSE | FALSE | FALSE | FALSE | TRUE | TRUE | TRUE |
| PCBP1 | 12 | TRUE | TRUE | FALSE | TRUE | TRUE | TRUE | TRUE | FALSE | FALSE | FALSE | TRUE | TRUE | TRUE | FALSE | FALSE | FALSE | TRUE | FALSE | TRUE | FALSE | TRUE |
| PCBP2 | 10 | TRUE | FALSE | FALSE | FALSE | TRUE | FALSE | FALSE | FALSE | TRUE | TRUE | FALSE | TRUE | TRUE | TRUE | FALSE | FALSE | FALSE | TRUE | TRUE | FALSE | TRUE |
| POLDIP3 | 8 | TRUE | TRUE | FALSE | FALSE | TRUE | FALSE | FALSE | FALSE | TRUE | FALSE | TRUE | FALSE | FALSE | FALSE | FALSE | FALSE | FALSE | FALSE | TRUE | TRUE | TRUE |
| PRDX6 | 7 | TRUE | FALSE | TRUE | FALSE | FALSE | FALSE | FALSE | FALSE | TRUE | FALSE | FALSE | TRUE | TRUE | TRUE | FALSE | FALSE | FALSE | FALSE | TRUE | FALSE | FALSE |
| PRMT1 | 7 | TRUE | FALSE | FALSE | FALSE | FALSE | FALSE | FALSE | FALSE | TRUE | TRUE | TRUE | TRUE | FALSE | FALSE | FALSE | FALSE | FALSE | TRUE | TRUE | FALSE | FALSE |
| PRPF19 | 6 | TRUE | FALSE | TRUE | FALSE | FALSE | FALSE | FALSE | FALSE | TRUE | FALSE | TRUE | FALSE | FALSE | FALSE | FALSE | FALSE | TRUE | FALSE | TRUE | FALSE | FALSE |
| RAB1B | 6 | TRUE | FALSE | TRUE | FALSE | FALSE | FALSE | FALSE | FALSE | TRUE | TRUE | FALSE | FALSE | FALSE | FALSE | FALSE | FALSE | TRUE | FALSE | TRUE | FALSE | FALSE |
| RAN | 8 | TRUE | FALSE | TRUE | FALSE | FALSE | FALSE | TRUE | FALSE | TRUE | FALSE | FALSE | TRUE | TRUE | FALSE | FALSE | FALSE | FALSE | FALSE | TRUE | FALSE | TRUE |
| RANBP1 | 4 | TRUE | FALSE | FALSE | FALSE | FALSE | FALSE | FALSE | FALSE | TRUE | FALSE | FALSE | FALSE | FALSE | FALSE | FALSE | FALSE | FALSE | TRUE | FALSE | TRUE | FALSE |
| RBMS1 | 5 | TRUE | FALSE | FALSE | FALSE | FALSE | TRUE | TRUE | FALSE | TRUE | FALSE | FALSE | FALSE | FALSE | FALSE | FALSE | FALSE | FALSE | FALSE | FALSE | TRUE | FALSE |
| RPL13 | 9 | TRUE | FALSE | TRUE | FALSE | TRUE | FALSE | FALSE | TRUE | TRUE | TRUE | TRUE | FALSE | FALSE | FALSE | FALSE | FALSE | FALSE | FALSE | TRUE | TRUE | FALSE |
| RPS2 | 11 | TRUE | FALSE | FALSE | FALSE | TRUE | TRUE | TRUE | TRUE | TRUE | TRUE | TRUE | FALSE | FALSE | FALSE | TRUE | FALSE | FALSE | FALSE | TRUE | TRUE | FALSE |
| RPS27A | 6 | TRUE | FALSE | FALSE | FALSE | FALSE | FALSE | FALSE | FALSE | TRUE | TRUE | FALSE | FALSE | FALSE | FALSE | FALSE | FALSE | TRUE | FALSE | TRUE | TRUE | FALSE |
| RPS3 | 11 | TRUE | FALSE | FALSE | FALSE | TRUE | TRUE | TRUE | TRUE | TRUE | TRUE | TRUE | FALSE | FALSE | FALSE | FALSE | FALSE | FALSE | FALSE | TRUE | TRUE | TRUE |
| RTCB | 11 | TRUE | TRUE | FALSE | FALSE | TRUE | FALSE | TRUE | TRUE | TRUE | TRUE | TRUE | FALSE | FALSE | FALSE | FALSE | FALSE | FALSE | FALSE | TRUE | TRUE | TRUE |
| SND1 | 15 | TRUE | TRUE | TRUE | TRUE | TRUE | TRUE | TRUE | TRUE | TRUE | TRUE | TRUE | TRUE | FALSE | FALSE | FALSE | FALSE | FALSE | FALSE | TRUE | TRUE | TRUE |
| SSB | 7 | TRUE | FALSE | FALSE | FALSE | TRUE | FALSE | FALSE | FALSE | TRUE | FALSE | TRUE | TRUE | TRUE | FALSE | TRUE | FALSE | FALSE | FALSE | FALSE | FALSE | FALSE |
| STRAP | 9 | TRUE | FALSE | FALSE | FALSE | FALSE | FALSE | FALSE | TRUE | TRUE | TRUE | FALSE | FALSE | TRUE | FALSE | FALSE | FALSE | TRUE | FALSE | TRUE | TRUE | TRUE |
| TKT | 6 | TRUE | FALSE | TRUE | FALSE | FALSE | FALSE | FALSE | FALSE | TRUE | FALSE | TRUE | TRUE | FALSE | FALSE | FALSE | FALSE | FALSE | FALSE | TRUE | FALSE | FALSE |
| TUBB6 | 4 | TRUE | FALSE | FALSE | FALSE | FALSE | FALSE | FALSE | FALSE | FALSE | FALSE | FALSE | TRUE | TRUE | FALSE | FALSE | FALSE | TRUE | FALSE | FALSE | FALSE | FALSE |
| USP10 | 10 | TRUE | FALSE | TRUE | TRUE | TRUE | FALSE | TRUE | FALSE | TRUE | TRUE | FALSE | FALSE | FALSE | FALSE | FALSE | FALSE | FALSE | FALSE | TRUE | TRUE | TRUE |
| YBX3 | 12 | TRUE | TRUE | FALSE | FALSE | TRUE | TRUE | TRUE | TRUE | TRUE | TRUE | FALSE | FALSE | FALSE | FALSE | FALSE | FALSE | FALSE | TRUE | TRUE | TRUE | TRUE |
| YWHAB | 7 | TRUE | FALSE | FALSE | FALSE | FALSE | FALSE | FALSE | FALSE | FALSE | FALSE | FALSE | TRUE | TRUE | TRUE | FALSE | FALSE | TRUE | TRUE | TRUE | FALSE | FALSE |
| ZC3HAV1 | 12 | TRUE | TRUE | TRUE | TRUE | TRUE | TRUE | FALSE | FALSE | TRUE | TRUE | TRUE | FALSE | FALSE | FALSE | TRUE | FALSE | FALSE | FALSE | TRUE | FALSE | TRUE |
| BCLAF1 | 6 | FALSE | TRUE | TRUE | FALSE | FALSE | FALSE | FALSE | FALSE | TRUE | FALSE | TRUE | FALSE | FALSE | FALSE | TRUE | FALSE | FALSE | FALSE | TRUE | FALSE | FALSE |
| CELF1 | 8 | FALSE | TRUE | FALSE | FALSE | TRUE | TRUE | TRUE | FALSE | TRUE | TRUE | TRUE | FALSE | FALSE | FALSE | FALSE | FALSE | FALSE | FALSE | FALSE | TRUE | FALSE |
| CHTOP | 8 | FALSE | TRUE | FALSE | FALSE | TRUE | FALSE | TRUE | FALSE | TRUE | TRUE | TRUE | FALSE | FALSE | FALSE | FALSE | FALSE | FALSE | FALSE | TRUE | TRUE | FALSE |
| CSE1L | 6 | FALSE | TRUE | FALSE | FALSE | FALSE | FALSE | FALSE | FALSE | TRUE | TRUE | TRUE | FALSE | FALSE | FALSE | FALSE | FALSE | FALSE | FALSE | TRUE | TRUE | FALSE |
| EIF3A | 12 | FALSE | TRUE | TRUE | TRUE | TRUE | TRUE | TRUE | FALSE | TRUE | TRUE | TRUE | FALSE | FALSE | FALSE | TRUE | FALSE | FALSE | FALSE | TRUE | TRUE | FALSE |
| EIF3C | 9 | FALSE | TRUE | FALSE | FALSE | TRUE | TRUE | TRUE | FALSE | TRUE | TRUE | TRUE | FALSE | FALSE | FALSE | FALSE | FALSE | FALSE | FALSE | TRUE | TRUE | FALSE |
| NPM1 | 12 | FALSE | TRUE | TRUE | FALSE | TRUE | TRUE | TRUE | FALSE | TRUE | FALSE | TRUE | FALSE | TRUE | FALSE | FALSE | FALSE | TRUE | TRUE | TRUE | TRUE | FALSE |
| PNN | 5 | FALSE | TRUE | FALSE | FALSE | FALSE | FALSE | FALSE | FALSE | TRUE | FALSE | TRUE | FALSE | FALSE | FALSE | FALSE | FALSE | FALSE | FALSE | TRUE | TRUE | FALSE |
| PRKDC | 9 | FALSE | TRUE | TRUE | TRUE | FALSE | FALSE | FALSE | FALSE | TRUE | TRUE | TRUE | FALSE | FALSE | FALSE | FALSE | FALSE | FALSE | FALSE | TRUE | TRUE | TRUE |
| RBM12B | 5 | FALSE | TRUE | TRUE | TRUE | FALSE | FALSE | FALSE | FALSE | TRUE | FALSE | TRUE | FALSE | FALSE | FALSE | FALSE | FALSE | FALSE | FALSE | FALSE | FALSE | FALSE |
| SAFB2 | 7 | FALSE | TRUE | TRUE | TRUE | TRUE | TRUE | FALSE | FALSE | TRUE | FALSE | TRUE | FALSE | FALSE | FALSE | FALSE | FALSE | FALSE | FALSE | FALSE | FALSE | FALSE |
| SF1 | 5 | FALSE | TRUE | FALSE | FALSE | TRUE | FALSE | FALSE | FALSE | TRUE | FALSE | TRUE | FALSE | FALSE | FALSE | FALSE | FALSE | FALSE | FALSE | FALSE | TRUE | FALSE |
| SLTM | 7 | FALSE | TRUE | TRUE | FALSE | TRUE | FALSE | FALSE | FALSE | TRUE | FALSE | TRUE | FALSE | FALSE | FALSE | TRUE | FALSE | FALSE | FALSE | TRUE | FALSE | FALSE |
| SNRNP200 | 8 | FALSE | TRUE | TRUE | TRUE | FALSE | FALSE | FALSE | FALSE | TRUE | TRUE | TRUE | FALSE | FALSE | FALSE | FALSE | FALSE | FALSE | FALSE | TRUE | TRUE | FALSE |
| THRAP3 | 7 | FALSE | TRUE | TRUE | FALSE | TRUE | TRUE | FALSE | FALSE | TRUE | FALSE | TRUE | FALSE | FALSE | FALSE | FALSE | FALSE | FALSE | FALSE | TRUE | FALSE | FALSE |
| TIAL1 | 9 | FALSE | TRUE | FALSE | FALSE | TRUE | TRUE | TRUE | FALSE | TRUE | TRUE | TRUE | FALSE | FALSE | FALSE | TRUE | FALSE | FALSE | FALSE | FALSE | TRUE | FALSE |
| TLN1 | 7 | FALSE | TRUE | TRUE | TRUE | FALSE | FALSE | FALSE | FALSE | TRUE | TRUE | TRUE | FALSE | FALSE | FALSE | FALSE | FALSE | FALSE | FALSE | TRUE | FALSE | FALSE |
| U2AF2 | 8 | FALSE | TRUE | FALSE | FALSE | TRUE | FALSE | FALSE | FALSE | TRUE | TRUE | TRUE | FALSE | FALSE | FALSE | TRUE | FALSE | FALSE | FALSE | TRUE | TRUE | FALSE |
| XRN2 | 7 | FALSE | TRUE | TRUE | FALSE | TRUE | FALSE | FALSE | FALSE | TRUE | TRUE | TRUE | FALSE | FALSE | FALSE | FALSE | FALSE | FALSE | FALSE | TRUE | FALSE | FALSE |
| YLPM1 | 8 | FALSE | TRUE | TRUE | TRUE | FALSE | FALSE | FALSE | FALSE | TRUE | TRUE | TRUE | FALSE | FALSE | FALSE | FALSE | FALSE | FALSE | FALSE | TRUE | TRUE | FALSE |
| ZFR | 10 | FALSE | TRUE | TRUE | TRUE | TRUE | FALSE | FALSE | FALSE | TRUE | TRUE | TRUE | FALSE | FALSE | FALSE | TRUE | FALSE | FALSE | FALSE | FALSE | TRUE | TRUE |
| DDX1 | 12 | FALSE | TRUE | TRUE | FALSE | TRUE | FALSE | TRUE | TRUE | TRUE | TRUE | TRUE | FALSE | FALSE | FALSE | FALSE | FALSE | FALSE | TRUE | TRUE | TRUE | TRUE |
| EIF3G | 5 | FALSE | TRUE | TRUE | FALSE | FALSE | FALSE | TRUE | FALSE | TRUE | FALSE | FALSE | FALSE | FALSE | FALSE | FALSE | FALSE | FALSE | FALSE | FALSE | TRUE | FALSE |
| KIF5B | 6 | FALSE | TRUE | TRUE | FALSE | FALSE | FALSE | FALSE | FALSE | TRUE | FALSE | FALSE | FALSE | TRUE | FALSE | FALSE | FALSE | FALSE | TRUE | TRUE | FALSE | FALSE |
| PDIA6 | 8 | FALSE | TRUE | FALSE | FALSE | TRUE | FALSE | FALSE | FALSE | TRUE | TRUE | FALSE | TRUE | FALSE | TRUE | FALSE | FALSE | FALSE | TRUE | TRUE | FALSE | FALSE |
| RPL26 | 5 | FALSE | FALSE | TRUE | FALSE | FALSE | TRUE | FALSE | FALSE | TRUE | TRUE | FALSE | FALSE | FALSE | FALSE | FALSE | FALSE | FALSE | FALSE | TRUE | FALSE | FALSE |
| RPS8 | 6 | FALSE | FALSE | TRUE | FALSE | TRUE | FALSE | FALSE | FALSE | TRUE | TRUE | TRUE | FALSE | FALSE | FALSE | FALSE | FALSE | FALSE | FALSE | TRUE | FALSE | FALSE |
| DDX21 | 7 | FALSE | FALSE | TRUE | FALSE | TRUE | FALSE | FALSE | FALSE | TRUE | FALSE | TRUE | FALSE | FALSE | FALSE | TRUE | FALSE | FALSE | FALSE | TRUE | TRUE | FALSE |
| UBA1 | 6 | FALSE | FALSE | TRUE | TRUE | FALSE | FALSE | FALSE | FALSE | TRUE | FALSE | TRUE | FALSE | FALSE | FALSE | FALSE | FALSE | FALSE | FALSE | TRUE | FALSE | TRUE |
| RPL4 | 7 | FALSE | FALSE | TRUE | FALSE | TRUE | FALSE | FALSE | FALSE | TRUE | TRUE | TRUE | TRUE | FALSE | FALSE | FALSE | FALSE | FALSE | FALSE | TRUE | FALSE | FALSE |
| RPL5 | 7 | FALSE | FALSE | TRUE | FALSE | TRUE | TRUE | FALSE | FALSE | TRUE | TRUE | TRUE | FALSE | FALSE | FALSE | FALSE | FALSE | FALSE | FALSE | TRUE | FALSE | FALSE |
| EIF5B | 5 | FALSE | FALSE | TRUE | FALSE | TRUE | FALSE | FALSE | FALSE | TRUE | FALSE | FALSE | FALSE | FALSE | FALSE | FALSE | FALSE | FALSE | FALSE | TRUE | TRUE | FALSE |
| UBAP2L | 11 | FALSE | FALSE | TRUE | FALSE | TRUE | TRUE | TRUE | FALSE | TRUE | TRUE | TRUE | FALSE | FALSE | FALSE | TRUE | FALSE | FALSE | FALSE | TRUE | TRUE | TRUE |
| MYH9 | 8 | FALSE | FALSE | TRUE | TRUE | TRUE | FALSE | TRUE | FALSE | TRUE | TRUE | TRUE | FALSE | FALSE | FALSE | FALSE | FALSE | FALSE | FALSE | TRUE | FALSE | FALSE |
| TRIM25 | 4 | FALSE | FALSE | TRUE | FALSE | TRUE | TRUE | FALSE | FALSE | FALSE | TRUE | FALSE | FALSE | FALSE | FALSE | FALSE | FALSE | FALSE | FALSE | FALSE | FALSE | FALSE |
| EEF2 | 7 | FALSE | FALSE | TRUE | FALSE | TRUE | FALSE | FALSE | FALSE | TRUE | TRUE | FALSE | FALSE | FALSE | FALSE | TRUE | FALSE | FALSE | FALSE | TRUE | FALSE | TRUE |
| RPL7 | 6 | FALSE | FALSE | TRUE | FALSE | TRUE | FALSE | FALSE | FALSE | TRUE | TRUE | TRUE | FALSE | FALSE | FALSE | FALSE | FALSE | FALSE | FALSE | TRUE | FALSE | FALSE |
| ESYT1 | 5 | FALSE | FALSE | TRUE | FALSE | TRUE | FALSE | FALSE | FALSE | TRUE | TRUE | FALSE | FALSE | FALSE | FALSE | FALSE | FALSE | FALSE | FALSE | TRUE | FALSE | FALSE |
| TOP2A | 5 | FALSE | FALSE | TRUE | TRUE | TRUE | FALSE | FALSE | FALSE | TRUE | FALSE | FALSE | FALSE | FALSE | FALSE | FALSE | FALSE | FALSE | FALSE | TRUE | FALSE | FALSE |
| RPS7 | 6 | FALSE | FALSE | TRUE | FALSE | TRUE | FALSE | FALSE | FALSE | TRUE | TRUE | TRUE | FALSE | FALSE | FALSE | FALSE | FALSE | FALSE | FALSE | TRUE | FALSE | FALSE |
| GANAB | 6 | FALSE | FALSE | TRUE | TRUE | TRUE | FALSE | FALSE | FALSE | TRUE | TRUE | FALSE | FALSE | FALSE | FALSE | FALSE | FALSE | FALSE | FALSE | TRUE | FALSE | FALSE |
| SUPT16H | 5 | FALSE | FALSE | TRUE | TRUE | TRUE | FALSE | FALSE | FALSE | TRUE | FALSE | FALSE | FALSE | FALSE | FALSE | FALSE | FALSE | FALSE | FALSE | TRUE | FALSE | FALSE |
| ADAR | 9 | FALSE | FALSE | TRUE | TRUE | TRUE | FALSE | TRUE | FALSE | TRUE | TRUE | TRUE | FALSE | FALSE | FALSE | FALSE | FALSE | FALSE | FALSE | TRUE | FALSE | TRUE |
| POLR2B | 4 | FALSE | FALSE | TRUE | FALSE | TRUE | FALSE | FALSE | FALSE | TRUE | FALSE | FALSE | FALSE | FALSE | FALSE | FALSE | FALSE | FALSE | FALSE | TRUE | FALSE | FALSE |
| DHX15 | 7 | FALSE | FALSE | TRUE | TRUE | TRUE | FALSE | FALSE | FALSE | TRUE | TRUE | TRUE | FALSE | FALSE | FALSE | FALSE | FALSE | FALSE | FALSE | TRUE | FALSE | FALSE |
| UPF1 | 11 | FALSE | FALSE | TRUE | TRUE | TRUE | TRUE | FALSE | TRUE | TRUE | TRUE | TRUE | FALSE | FALSE | FALSE | FALSE | FALSE | FALSE | FALSE | TRUE | TRUE | TRUE |
| FLNA | 8 | FALSE | FALSE | TRUE | FALSE | FALSE | FALSE | FALSE | FALSE | TRUE | FALSE | TRUE | TRUE | TRUE | TRUE | FALSE | FALSE | FALSE | FALSE | TRUE | FALSE | TRUE |
| RPL6 | 8 | FALSE | FALSE | TRUE | FALSE | TRUE | FALSE | FALSE | TRUE | TRUE | TRUE | TRUE | FALSE | FALSE | FALSE | FALSE | FALSE | FALSE | TRUE | TRUE | FALSE | FALSE |
| RPL3 | 8 | FALSE | FALSE | TRUE | FALSE | TRUE | TRUE | FALSE | FALSE | TRUE | TRUE | TRUE | FALSE | FALSE | FALSE | FALSE | FALSE | FALSE | FALSE | TRUE | TRUE | FALSE |
| LARP1 | 10 | FALSE | FALSE | TRUE | TRUE | TRUE | TRUE | FALSE | FALSE | TRUE | TRUE | TRUE | FALSE | FALSE | FALSE | TRUE | FALSE | FALSE | FALSE | TRUE | TRUE | FALSE |
| HSP90AB1 | 6 | FALSE | FALSE | TRUE | FALSE | FALSE | FALSE | TRUE | FALSE | FALSE | FALSE | FALSE | FALSE | FALSE | FALSE | FALSE | FALSE | TRUE | FALSE | TRUE | TRUE | TRUE |
| PRPF8 | 8 | FALSE | FALSE | TRUE | TRUE | TRUE | FALSE | FALSE | FALSE | TRUE | TRUE | TRUE | FALSE | FALSE | FALSE | FALSE | FALSE | FALSE | FALSE | TRUE | TRUE | FALSE |
| PARP1 | 10 | FALSE | FALSE | TRUE | TRUE | TRUE | FALSE | FALSE | FALSE | TRUE | TRUE | TRUE | TRUE | TRUE | FALSE | FALSE | FALSE | FALSE | FALSE | TRUE | FALSE | TRUE |
| XRCC5 | 9 | FALSE | FALSE | TRUE | FALSE | TRUE | FALSE | FALSE | FALSE | TRUE | TRUE | TRUE | TRUE | FALSE | FALSE | TRUE | FALSE | FALSE | FALSE | TRUE | FALSE | TRUE |
| ABCF1 | 7 | FALSE | FALSE | TRUE | TRUE | TRUE | FALSE | FALSE | FALSE | TRUE | TRUE | TRUE | FALSE | FALSE | FALSE | FALSE | FALSE | FALSE | FALSE | FALSE | TRUE | FALSE |
| EIF4G1 | 11 | FALSE | FALSE | TRUE | FALSE | TRUE | TRUE | TRUE | FALSE | TRUE | TRUE | TRUE | FALSE | FALSE | FALSE | FALSE | FALSE | FALSE | TRUE | TRUE | TRUE | TRUE |
| YTHDF2 | 6 | FALSE | FALSE | TRUE | FALSE | FALSE | FALSE | FALSE | TRUE | TRUE | TRUE | TRUE | FALSE | FALSE | FALSE | FALSE | FALSE | FALSE | FALSE | FALSE | TRUE | FALSE |
| RBM15 | 5 | FALSE | FALSE | TRUE | TRUE | TRUE | FALSE | FALSE | FALSE | TRUE | FALSE | TRUE | FALSE | FALSE | FALSE | FALSE | FALSE | FALSE | FALSE | FALSE | FALSE | FALSE |
| RPS6 | 7 | FALSE | FALSE | TRUE | FALSE | TRUE | TRUE | FALSE | FALSE | TRUE | TRUE | TRUE | FALSE | FALSE | FALSE | FALSE | FALSE | FALSE | FALSE | TRUE | FALSE | FALSE |
| ATXN2L | 10 | FALSE | FALSE | TRUE | FALSE | TRUE | TRUE | TRUE | FALSE | TRUE | TRUE | TRUE | FALSE | FALSE | FALSE | FALSE | FALSE | FALSE | FALSE | TRUE | TRUE | TRUE |
| PRRC2C | 8 | FALSE | FALSE | TRUE | TRUE | TRUE | FALSE | TRUE | FALSE | TRUE | TRUE | FALSE | FALSE | FALSE | FALSE | FALSE | FALSE | FALSE | FALSE | TRUE | TRUE | FALSE |
| XRCC6 | 12 | FALSE | FALSE | TRUE | TRUE | TRUE | FALSE | FALSE | FALSE | TRUE | TRUE | TRUE | TRUE | TRUE | FALSE | TRUE | FALSE | FALSE | FALSE | TRUE | TRUE | TRUE |
| RPS16 | 6 | FALSE | FALSE | TRUE | FALSE | TRUE | FALSE | FALSE | FALSE | TRUE | TRUE | FALSE | FALSE | FALSE | FALSE | FALSE | FALSE | FALSE | FALSE | TRUE | FALSE | TRUE |
| HSPA8 | 4 | FALSE | FALSE | TRUE | FALSE | FALSE | FALSE | TRUE | FALSE | FALSE | FALSE | FALSE | FALSE | FALSE | FALSE | FALSE | FALSE | TRUE | FALSE | TRUE | FALSE | FALSE |
| RPA1 | 5 | FALSE | FALSE | TRUE | FALSE | TRUE | FALSE | FALSE | FALSE | TRUE | FALSE | TRUE | FALSE | FALSE | FALSE | FALSE | FALSE | FALSE | FALSE | TRUE | FALSE | FALSE |
| NONO | 11 | FALSE | FALSE | TRUE | FALSE | TRUE | TRUE | FALSE | FALSE | TRUE | TRUE | TRUE | FALSE | FALSE | FALSE | TRUE | FALSE | TRUE | FALSE | TRUE | TRUE | TRUE |
| EEF1G | 6 | FALSE | FALSE | TRUE | TRUE | TRUE | FALSE | FALSE | FALSE | TRUE | TRUE | FALSE | FALSE | FALSE | FALSE | FALSE | FALSE | FALSE | FALSE | TRUE | FALSE | FALSE |
| ATP1A1 | 7 | FALSE | FALSE | TRUE | FALSE | TRUE | FALSE | FALSE | FALSE | TRUE | FALSE | FALSE | TRUE | TRUE | FALSE | FALSE | FALSE | TRUE | FALSE | TRUE | FALSE | FALSE |
| RPL7A | 6 | FALSE | FALSE | TRUE | FALSE | TRUE | FALSE | FALSE | FALSE | TRUE | TRUE | TRUE | FALSE | FALSE | FALSE | FALSE | FALSE | FALSE | FALSE | TRUE | FALSE | FALSE |
| MKI67 | 5 | FALSE | FALSE | FALSE | TRUE | TRUE | FALSE | FALSE | FALSE | TRUE | FALSE | FALSE | FALSE | FALSE | FALSE | FALSE | FALSE | FALSE | FALSE | TRUE | TRUE | FALSE |
| KPNB1 | 7 | FALSE | FALSE | FALSE | TRUE | FALSE | FALSE | FALSE | FALSE | TRUE | FALSE | FALSE | TRUE | TRUE | FALSE | FALSE | FALSE | FALSE | FALSE | TRUE | TRUE | TRUE |
| PRRC2A | 9 | FALSE | FALSE | FALSE | TRUE | TRUE | FALSE | TRUE | FALSE | TRUE | TRUE | TRUE | FALSE | FALSE | FALSE | FALSE | FALSE | FALSE | FALSE | TRUE | TRUE | TRUE |
| DHX57 | 5 | FALSE | FALSE | FALSE | TRUE | FALSE | FALSE | TRUE | FALSE | TRUE | TRUE | FALSE | FALSE | FALSE | FALSE | FALSE | FALSE | FALSE | FALSE | FALSE | TRUE | FALSE |
| SSBP1 | 7 | FALSE | FALSE | FALSE | FALSE | TRUE | FALSE | FALSE | FALSE | FALSE | FALSE | FALSE | TRUE | TRUE | TRUE | FALSE | TRUE | TRUE | FALSE | TRUE | FALSE | FALSE |
| TOP1 | 4 | FALSE | FALSE | FALSE | FALSE | TRUE | FALSE | FALSE | FALSE | TRUE | FALSE | FALSE | FALSE | FALSE | FALSE | FALSE | FALSE | FALSE | FALSE | TRUE | TRUE | FALSE |
| SERBP1 | 10 | FALSE | FALSE | FALSE | FALSE | TRUE | TRUE | FALSE | FALSE | TRUE | TRUE | TRUE | FALSE | FALSE | FALSE | TRUE | TRUE | FALSE | FALSE | TRUE | TRUE | TRUE |
| RBM39 | 7 | FALSE | FALSE | FALSE | FALSE | TRUE | FALSE | FALSE | FALSE | TRUE | TRUE | TRUE | FALSE | FALSE | FALSE | TRUE | FALSE | FALSE | FALSE | TRUE | TRUE | FALSE |
| RPL10 | 5 | FALSE | FALSE | FALSE | FALSE | TRUE | FALSE | FALSE | FALSE | TRUE | FALSE | TRUE | FALSE | FALSE | FALSE | FALSE | FALSE | FALSE | FALSE | TRUE | TRUE | FALSE |
| RPS18 | 6 | FALSE | FALSE | FALSE | FALSE | TRUE | FALSE | FALSE | FALSE | TRUE | TRUE | TRUE | TRUE | FALSE | FALSE | FALSE | FALSE | FALSE | FALSE | TRUE | FALSE | FALSE |
| RPSA | 6 | FALSE | FALSE | FALSE | FALSE | TRUE | FALSE | FALSE | FALSE | TRUE | TRUE | FALSE | TRUE | FALSE | FALSE | FALSE | FALSE | FALSE | TRUE | TRUE | FALSE | FALSE |
| EIF3D | 6 | FALSE | FALSE | FALSE | FALSE | TRUE | TRUE | TRUE | FALSE | TRUE | FALSE | TRUE | FALSE | FALSE | FALSE | FALSE | FALSE | FALSE | FALSE | FALSE | TRUE | FALSE |
| TMPO | 4 | FALSE | FALSE | FALSE | FALSE | TRUE | FALSE | FALSE | FALSE | TRUE | FALSE | FALSE | FALSE | FALSE | FALSE | FALSE | FALSE | FALSE | FALSE | TRUE | TRUE | FALSE |
| DDX46 | 5 | FALSE | FALSE | FALSE | FALSE | TRUE | FALSE | FALSE | FALSE | TRUE | FALSE | TRUE | FALSE | FALSE | FALSE | FALSE | FALSE | FALSE | FALSE | TRUE | TRUE | FALSE |
| RBM3 | 9 | FALSE | FALSE | FALSE | FALSE | TRUE | TRUE | TRUE | FALSE | TRUE | TRUE | TRUE | TRUE | TRUE | FALSE | FALSE | TRUE | FALSE | FALSE | FALSE | FALSE | FALSE |
| CKAP4 | 4 | FALSE | FALSE | FALSE | FALSE | TRUE | FALSE | FALSE | FALSE | TRUE | TRUE | FALSE | FALSE | FALSE | FALSE | FALSE | FALSE | FALSE | FALSE | FALSE | TRUE | FALSE |
| HSPA1B | 5 | FALSE | FALSE | FALSE | FALSE | TRUE | FALSE | FALSE | FALSE | TRUE | FALSE | FALSE | TRUE | FALSE | TRUE | FALSE | FALSE | FALSE | FALSE | TRUE | FALSE | FALSE |
| SON | 4 | FALSE | FALSE | FALSE | FALSE | TRUE | FALSE | FALSE | FALSE | TRUE | FALSE | FALSE | FALSE | FALSE | FALSE | FALSE | FALSE | FALSE | FALSE | TRUE | TRUE | FALSE |
| GIGYF2 | 4 | FALSE | FALSE | FALSE | FALSE | TRUE | FALSE | FALSE | FALSE | TRUE | TRUE | FALSE | FALSE | FALSE | FALSE | FALSE | FALSE | FALSE | FALSE | FALSE | TRUE | FALSE |
| LRRC47 | 6 | FALSE | FALSE | FALSE | FALSE | TRUE | TRUE | FALSE | FALSE | TRUE | TRUE | FALSE | FALSE | FALSE | FALSE | FALSE | FALSE | FALSE | FALSE | TRUE | TRUE | FALSE |
| LARP4B | 6 | FALSE | FALSE | FALSE | FALSE | TRUE | FALSE | TRUE | FALSE | TRUE | TRUE | FALSE | FALSE | FALSE | FALSE | FALSE | FALSE | FALSE | FALSE | FALSE | TRUE | TRUE |
| ZC3H7B | 3 | FALSE | FALSE | FALSE | FALSE | TRUE | FALSE | FALSE | FALSE | TRUE | FALSE | FALSE | FALSE | FALSE | FALSE | FALSE | FALSE | FALSE | FALSE | FALSE | TRUE | FALSE |
| RPS13 | 6 | FALSE | FALSE | FALSE | FALSE | TRUE | FALSE | FALSE | FALSE | TRUE | TRUE | TRUE | FALSE | FALSE | FALSE | FALSE | FALSE | FALSE | FALSE | TRUE | TRUE | FALSE |
| FAM98A | 8 | FALSE | FALSE | FALSE | FALSE | TRUE | TRUE | TRUE | FALSE | TRUE | TRUE | FALSE | FALSE | FALSE | FALSE | TRUE | FALSE | FALSE | FALSE | FALSE | TRUE | TRUE |
| PUM2 | 5 | FALSE | FALSE | FALSE | FALSE | TRUE | TRUE | TRUE | FALSE | FALSE | TRUE | FALSE | FALSE | FALSE | FALSE | FALSE | FALSE | FALSE | FALSE | FALSE | TRUE | FALSE |
| PUF60 | 6 | FALSE | FALSE | FALSE | FALSE | TRUE | FALSE | FALSE | FALSE | TRUE | FALSE | TRUE | FALSE | FALSE | FALSE | FALSE | FALSE | TRUE | FALSE | TRUE | TRUE | FALSE |
| ATXN2 | 6 | FALSE | FALSE | FALSE | FALSE | TRUE | TRUE | TRUE | FALSE | TRUE | TRUE | FALSE | FALSE | FALSE | FALSE | FALSE | FALSE | FALSE | FALSE | FALSE | TRUE | FALSE |
| SF3B1 | 6 | FALSE | FALSE | FALSE | FALSE | TRUE | FALSE | FALSE | FALSE | TRUE | TRUE | TRUE | FALSE | FALSE | FALSE | FALSE | FALSE | FALSE | FALSE | TRUE | TRUE | FALSE |
| FXR2 | 7 | FALSE | FALSE | FALSE | FALSE | TRUE | TRUE | TRUE | FALSE | TRUE | TRUE | FALSE | FALSE | FALSE | FALSE | TRUE | FALSE | FALSE | FALSE | FALSE | TRUE | FALSE |
| SRP72 | 5 | FALSE | FALSE | FALSE | FALSE | TRUE | FALSE | FALSE | FALSE | TRUE | TRUE | FALSE | FALSE | FALSE | FALSE | FALSE | FALSE | FALSE | FALSE | TRUE | TRUE | FALSE |
| LARP4 | 7 | FALSE | FALSE | FALSE | FALSE | TRUE | FALSE | TRUE | TRUE | TRUE | TRUE | TRUE | FALSE | FALSE | FALSE | FALSE | FALSE | FALSE | FALSE | FALSE | TRUE | FALSE |
| SF3B2 | 6 | FALSE | FALSE | FALSE | FALSE | TRUE | FALSE | TRUE | FALSE | TRUE | FALSE | TRUE | FALSE | FALSE | FALSE | FALSE | FALSE | FALSE | FALSE | TRUE | TRUE | FALSE |
| PURB | 5 | FALSE | FALSE | FALSE | FALSE | TRUE | FALSE | TRUE | FALSE | TRUE | TRUE | FALSE | FALSE | FALSE | FALSE | FALSE | FALSE | FALSE | FALSE | FALSE | TRUE | FALSE |
| CAPRIN1 | 9 | FALSE | FALSE | FALSE | FALSE | FALSE | FALSE | TRUE | TRUE | TRUE | TRUE | TRUE | FALSE | FALSE | FALSE | FALSE | FALSE | FALSE | TRUE | TRUE | TRUE | TRUE |
| LSM14A | 5 | FALSE | FALSE | FALSE | FALSE | FALSE | FALSE | TRUE | TRUE | TRUE | TRUE | FALSE | FALSE | FALSE | FALSE | FALSE | FALSE | FALSE | FALSE | FALSE | TRUE | FALSE |
| SPATS2L | 4 | FALSE | FALSE | FALSE | FALSE | FALSE | FALSE | TRUE | FALSE | TRUE | TRUE | FALSE | FALSE | FALSE | FALSE | FALSE | FALSE | FALSE | FALSE | FALSE | TRUE | FALSE |
| AGO2 | 4 | FALSE | FALSE | FALSE | FALSE | FALSE | FALSE | TRUE | FALSE | FALSE | TRUE | TRUE | FALSE | FALSE | FALSE | FALSE | FALSE | FALSE | FALSE | FALSE | TRUE | FALSE |
| NUFIP2 | 6 | FALSE | FALSE | FALSE | FALSE | FALSE | FALSE | TRUE | FALSE | TRUE | TRUE | FALSE | FALSE | FALSE | FALSE | FALSE | FALSE | FALSE | FALSE | TRUE | TRUE | TRUE |
| YTHDC2 | 3 | FALSE | FALSE | FALSE | FALSE | FALSE | FALSE | TRUE | FALSE | FALSE | TRUE | FALSE | FALSE | FALSE | FALSE | FALSE | FALSE | FALSE | FALSE | FALSE | TRUE | FALSE |
| KPNA2 | 5 | FALSE | FALSE | FALSE | FALSE | FALSE | FALSE | FALSE | FALSE | TRUE | FALSE | FALSE | FALSE | FALSE | FALSE | TRUE | FALSE | FALSE | FALSE | TRUE | TRUE | TRUE |

Supplementary Table 3

| **Protein** | **Virus-linked** | **GO annotation “RNA binding”** | **RBD classification** |
| --- | --- | --- | --- |
| FAM120A | FALSE | TRUE | other |
| FUBP1 | TRUE | TRUE | classical |
| HDLBP | FALSE | TRUE | classical |
| HNRNPA1 | TRUE | TRUE | classical |
| HNRNPA2B1 | TRUE | TRUE | classical |
| HNRNPA3 | FALSE | TRUE | classical |
| HNRNPAB | FALSE | TRUE | classical |
| HNRNPC | TRUE | TRUE | classical |
| HNRNPH1 | FALSE | TRUE | classical |
| HNRNPK | TRUE | TRUE | classical |
| HNRNPL | FALSE | TRUE | classical |
| HNRNPM | FALSE | TRUE | classical |
| IGF2BP1 | TRUE | TRUE | classical |
| IGF2BP3 | TRUE | TRUE | classical |
| L1RE1 | FALSE | FALSE | other |
| MATR3 | TRUE | TRUE | classical |
| PABPC1 | TRUE | TRUE | classical |
| PTBP1 | TRUE | TRUE | classical |
| PURA | TRUE | TRUE | other |
| RACK1 | TRUE | TRUE | nonclassical |
| RPL15 | TRUE | TRUE | nonclassical |
| RPL24 | TRUE | TRUE | nonclassical |
| RPS14 | TRUE | TRUE | nonclassical |
| RRBP1 | FALSE | TRUE | other |
| SFPQ | TRUE | TRUE | classical |
| SRSF2 | TRUE | TRUE | classical |
| SRSF6 | TRUE | TRUE | classical |
| TARDBP | TRUE | TRUE | classical |
| TIA1 | TRUE | TRUE | classical |
| YBX1 | TRUE | TRUE | classical |
| ZCCHC3 | TRUE | TRUE | nonclassical |
| ZNF638 | FALSE | TRUE | classical |
| ACLY | TRUE | FALSE | other |
| AHCY | FALSE | FALSE | other |
| ATIC | FALSE | FALSE | other |
| C1QBP | TRUE | TRUE | other |
| CNBP | TRUE | TRUE | nonclassical |
| CNOT1 | FALSE | TRUE | other |
| COPB2 | FALSE | FALSE | nonclassical |
| CSDE1 | TRUE | TRUE | classical |
| DDX3X | TRUE | TRUE | classical |
| DHX9 | TRUE | TRUE | classical |
| EIF2S1 | TRUE | TRUE | nonclassical |
| EIF4A1 | TRUE | TRUE | classical |
| EIF4B | TRUE | TRUE | classical |
| EIF4H | TRUE | TRUE | classical |
| EZR | FALSE | TRUE | nonclassical |
| FEN1 | TRUE | FALSE | other |
| FUBP3 | FALSE | TRUE | classical |
| FXR1 | TRUE | TRUE | classical |
| G3BP1 | TRUE | TRUE | classical |
| GDI2 | FALSE | TRUE | other |
| HNRNPD | TRUE | TRUE | classical |
| HNRNPDL | FALSE | TRUE | classical |
| HNRNPU | TRUE | TRUE | nonclassical |
| HSPA4 | TRUE | FALSE | nonclassical |
| IGF2BP2 | FALSE | TRUE | classical |
| ILF3 | TRUE | TRUE | classical |
| MAP4 | TRUE | TRUE | nonclassical |
| MOV10 | TRUE | TRUE | classical |
| NCL | TRUE | TRUE | classical |
| PABPC4 | TRUE | TRUE | classical |
| PCBP1 | TRUE | TRUE | classical |
| PCBP2 | TRUE | TRUE | classical |
| POLDIP3 | FALSE | TRUE | classical |
| PRDX6 | TRUE | FALSE | other |
| PRMT1 | TRUE | TRUE | other |
| PRPF19 | FALSE | FALSE | nonclassical |
| RAB1B | TRUE | FALSE | other |
| RAN | TRUE | TRUE | other |
| RANBP1 | TRUE | FALSE | other |
| RBMS1 | FALSE | TRUE | classical |
| RPL13 | TRUE | TRUE | nonclassical |
| RPS2 | TRUE | TRUE | nonclassical |
| RPS27A | TRUE | TRUE | nonclassical |
| RPS3 | TRUE | TRUE | classical |
| RTCB | FALSE | TRUE | nonclassical |
| SND1 | TRUE | TRUE | nonclassical |
| SSB | TRUE | TRUE | classical |
| STRAP | TRUE | TRUE | nonclassical |
| TKT | TRUE | FALSE | other |
| TUBB6 | FALSE | FALSE | other |
| USP10 | TRUE | TRUE | other |
| YBX3 | TRUE | TRUE | classical |
| YWHAB | TRUE | FALSE | nonclassical |
| ZC3HAV1 | TRUE | TRUE | other |
| BCLAF1 | TRUE | TRUE | other |
| CELF1 | TRUE | TRUE | classical |
| CHTOP | FALSE | TRUE | other |
| CSE1L | FALSE | FALSE | other |
| EIF3A | TRUE | TRUE | other |
| EIF3C | TRUE | TRUE | other |
| NPM1 | TRUE | TRUE | other |
| PNN | TRUE | TRUE | other |
| PRKDC | TRUE | TRUE | other |
| RBM12B | FALSE | TRUE | classical |
| SAFB2 | FALSE | TRUE | classical |
| SF1 | TRUE | TRUE | classical |
| SLTM | FALSE | TRUE | classical |
| SNRNP200 | FALSE | TRUE | classical |
| THRAP3 | FALSE | TRUE | other |
| TIAL1 | TRUE | TRUE | classical |
| TLN1 | TRUE | FALSE | other |
| U2AF2 | TRUE | TRUE | classical |
| XRN2 | TRUE | TRUE | other |
| YLPM1 | TRUE | TRUE | other |
| ZFR | FALSE | TRUE | nonclassical |
| DDX1 | TRUE | TRUE | classical |
| EIF3G | TRUE | TRUE | classical |
| KIF5B | TRUE | FALSE | other |
| PDIA6 | FALSE | FALSE | nonclassical |
| SSBP1 | FALSE | TRUE | nonclassical |
| TOP1 | TRUE | TRUE | other |
| PARP1 | TRUE | TRUE | other |
| RPL5 | TRUE | TRUE | nonclassical |
| RPL6 | TRUE | TRUE | nonclassical |
| RPS7 | TRUE | TRUE | nonclassical |
| RPL4 | TRUE | TRUE | nonclassical |
| RPL7 | TRUE | TRUE | nonclassical |
| RPA1 | TRUE | FALSE | other |
| SUPT16H | TRUE | TRUE | other |
| RPL7A | TRUE | TRUE | nonclassical |
| RPL3 | TRUE | TRUE | nonclassical |
| TOP2A | TRUE | TRUE | other |
| SERBP1 | FALSE | TRUE | nonclassical |
| RBM39 | FALSE | TRUE | classical |
| RPS6 | TRUE | TRUE | nonclassical |
| EIF5B | TRUE | TRUE | nonclassical |
| XRCC6 | TRUE | TRUE | nonclassical |
| POLR2B | TRUE | TRUE | other |
| MKI67 | TRUE | TRUE | other |
| RPL10 | TRUE | TRUE | nonclassical |
| RPS18 | TRUE | TRUE | nonclassical |
| LARP1 | TRUE | TRUE | other |
| RPS8 | TRUE | TRUE | nonclassical |
| RPSA | TRUE | TRUE | nonclassical |
| EIF3D | TRUE | TRUE | other |
| TMPO | FALSE | FALSE | other |
| NONO | TRUE | TRUE | classical |
| ATXN2L | FALSE | TRUE | other |
| DDX46 | FALSE | TRUE | classical |
| RBM3 | TRUE | TRUE | classical |
| CKAP4 | TRUE | TRUE | other |
| HSPA1B | TRUE | TRUE | nonclassical |
| SON | TRUE | TRUE | other |
| GIGYF2 | FALSE | TRUE | other |
| LRRC47 | FALSE | TRUE | other |
| UPF1 | TRUE | TRUE | classical |
| PRRC2C | FALSE | TRUE | other |
| PRPF8 | FALSE | TRUE | classical |
| PRRC2A | FALSE | TRUE | other |
| LARP4B | FALSE | TRUE | other |
| ZC3H7B | TRUE | TRUE | classical |
| TRIM25 | TRUE | TRUE | other |
| RBM15 | TRUE | TRUE | classical |
| DDX21 | TRUE | TRUE | classical |
| EEF2 | TRUE | TRUE | nonclassical |
| ATP1A1 | TRUE | FALSE | other |
| RPS16 | TRUE | TRUE | nonclassical |
| EIF4G1 | TRUE | TRUE | other |
| MYH9 | TRUE | TRUE | other |
| RPS13 | TRUE | TRUE | nonclassical |
| XRCC5 | TRUE | TRUE | other |
| FAM98A | FALSE | TRUE | other |
| ABCF1 | FALSE | TRUE | other |
| UBAP2L | FALSE | TRUE | other |
| PUM2 | FALSE | TRUE | classical |
| PUF60 | FALSE | TRUE | classical |
| ATXN2 | TRUE | TRUE | other |
| SF3B1 | TRUE | TRUE | other |
| FXR2 | FALSE | TRUE | classical |
| SRP72 | FALSE | TRUE | other |
| EEF1G | TRUE | FALSE | other |
| LARP4 | FALSE | TRUE | other |
| ESYT1 | FALSE | FALSE | other |
| ADAR | TRUE | TRUE | classical |
| SF3B2 | TRUE | TRUE | nonclassical |
| PURB | TRUE | TRUE | other |
| DHX15 | TRUE | TRUE | classical |
| GANAB | FALSE | TRUE | other |
| RPL26 | TRUE | TRUE | nonclassical |
| CAPRIN1 | TRUE | TRUE | other |
| LSM14A | TRUE | TRUE | nonclassical |
| SPATS2L | FALSE | TRUE | other |
| AGO2 | TRUE | TRUE | classical |
| NUFIP2 | FALSE | TRUE | other |
| DHX57 | FALSE | TRUE | classical |
| HSP90AB1 | TRUE | TRUE | nonclassical |
| HSPA8 | TRUE | TRUE | nonclassical |
| YTHDC2 | TRUE | TRUE | classical |
| YTHDF2 | TRUE | TRUE | nonclassical |
| KPNB1 | TRUE | TRUE | other |
| FLNA | TRUE | TRUE | nonclassical |
| UBA1 | TRUE | TRUE | other |
| KPNA2 | TRUE | TRUE | other |

**Citations (as numbered in Review article)**

25 Kim, B. *et al.* (2020) Discovery of Widespread Host Protein Interactions with the Pre-replicated
Genome of CHIKV Using VIR-CLASP. *Mol. Cell* 78, 624-640

26 Kamel, W. *et al.* (2021) Global analysis of protein-RNA interactions in SARS-CoV-2-infected cells reveals key regulators of infection. *Mol. Cell* 81, 2851-2867.e7

27 Knoener, R.A. *et al.* (2021) Identification of host proteins differentially associated with HIV-1 RNA splice variants. *Elife* 10, 1–32

40 Knoener, R.A. *et al.* (2017) Elucidating the in vivo interactome of HIV-1 RNA by hybridization capture and mass spectrometry. *Sci. Rep.* 7, 1–16

41 Lee, S. et al. (2021) The SARS-CoV-2 RNA interactome, Mol. Cell, 81, 2838–2850.e6.

42 Phillips, S.L. *et al.* (2016) Identification of Proteins Bound to Dengue Viral RNA In Vivo Reveals New Host Proteins Important for Virus Replication. *MBio* 7, 1–10

43 Schmidt, N. et al. (2021) The SARS-CoV-2 RNA–protein interactome in infected human cells, Nat. Microbiol., 6, 339–353.

44 Ooi, Y.S. *et al.* (2019) An RNA-centric dissection of host complexes controlling flavivirus infection. *Nat. Microbiol.* 4, 2369–2382.

45 Labeau, A. et al. (2021) Characterization and functional interrogation of SARS-CoV-2 RNA interactome, bioRxiv, Published online March 23, 2021 https://doi.org/10.1101/2021.03.23.436611.

46 Flynn, R.A. et al. (2021) Discovery and functional interrogation of SARS-CoV- 2 RNA-host protein interactions, Cell, 184, 2021, 2394–2411.e16.

50 Lenarcic, E.M. *et al.* (2013) Thiouracil Cross-Linking Mass Spectrometry: a Cell-Based Method To Identify Host Factors Involved in Viral Amplification. *J. Virol.* 87, 8697–8712

51 Viktorovskaya, O.V. et al. (2016) Identification of RNA binding proteins associated with dengue virus RNA in infected cells reveals temporally distinct host factor requirements, PLoS Negl. Trop. Dis., 10, e0004921.

53 Gebhart, N.N. et al. (2020) Comparative analyses of alphaviral RNA: protein complexes reveals conserved host-pathogen interactions, PLoS One, 15, e0238254.
